# Implementing a Risk-Stratified National Breast Screening Programme in the United Kingdom: A Model-Based Cost-effectiveness Analysis

**DOI:** 10.1158/1055-9965.EPI-26-0103

**Published:** 2026-07-31

**Authors:** Stuart J. Wright, Joe Hilton, Gabriel Rogers, Ewan Gray, Anna Donten, Lorna McWilliams, D. Gareth R. Evans, David P. French, Katherine Payne

**Affiliations:** 1 Manchester Centre for Health Economics, Division of Population Health, Health Services Research and Primary Care, School of Health Sciences, Faculty of Biology, Medicine and Health, The University of Manchester, Manchester, United Kingdom.; 2 Manchester Breast Centre, Manchester Cancer Research Centre, University of Manchester, Manchester, United Kingdom.; 3 Manchester Biomedical Research Centre, The University of Manchester, Manchester, United Kingdom.; 4 Independent Health Economics Consultant, Edinburgh, United Kingdom.; 5 Division of Psychology and Mental Health, Manchester Centre for Health Psychology, School of Health Sciences, Faculty of Biology, Medicine, and Health, The University of Manchester, Manchester, United Kingdom.; 6 The Prevent Breast Cancer Research Unit, The Nightingale Centre, Manchester University NHS Foundation Trust, Manchester, United Kingdom.; 7 Division of Evolution and Genomic Science, The University of Manchester, Manchester, United Kingdom.; 8 Manchester Centre of Genomic Medicine, Manchester University NHS Foundation Trust, Manchester, United Kingdom.; 9 Manchester Academic Health Science Centre, Health innovation Manchester, Manchester, United Kingdom.; 10 Division of Cancer Sciences, School of Medical Sciences, Faculty of Biology, Medicine and Health, The University of Manchester, Manchester, United Kingdom.

## Abstract

**Background:**

Risk-stratified breast screening, in which women are offered different screening intervals based on their breast cancer risk, has been proposed as a way to improve the benefits of screening in the United Kingdom National Breast Screening Programme (UK-NBSP). The aim of this study was to explore the cost-effectiveness of potential risk-stratified breast screening strategies in the UK-NBSP.

**Methods:**

An existing validated discrete event simulation model (MANC-RISK-SCREEN) was used to evaluate the cost-effectiveness of 3 risk-stratified NBSPs (risk 1, risk 2, and risk 3) compared with the current UK-NBSP, 2-yearly screening, and no screening [lifetime horizon; health service perspective; costs (£; at 2024 prices)]. MANC-RISK-SCREEN was modified to account for bias in risk prediction and allocation to risk categories (risk misclassification). Uncertainty in the results due to model inputs was quantified using probabilistic sensitivity analysis and one-way sensitivity analyses.

**Results:**

At a cost-effectiveness threshold of £20,000 per quality-adjusted life year, 2-yearly screening was most likely to be cost-effective, followed by a risk-stratified approach whereby women at high risk (>8% 10-year risk) were screened annually and women at moderate risk (5%–8% 10-year risk) biannually. When risk misclassification was accounted for, the ranking of risk-stratified screening strategies improved relative to universal strategies.

**Conclusions:**

If 2-yearly screening is infeasible due to resource constraints, implementing a risk-stratified approach in the existing NBSP in the United Kingdom would be a cost-effective use of healthcare resources.

**Impact:**

Implementation of risk-stratified breast screening in the UK national screening program has the potential to improve cancer detection at a reasonable additional resource use and cost.

## Introduction

The current National Breast Screening Programme (NBSP) in the United Kingdom offers a breast x-ray (mammogram) every 3 years to women between 50 and 70 years of age. This approach to delivering an NBSP assumes that all women are at equal risk of developing breast cancer (1). There is a developing body of evidence that supports the use of risk prediction tools, such as the Tyrer–Cuzick questionnaire, to define an individual woman’s 10-year and lifetime risk of developing breast cancer (2, 3). The population of women eligible for breast screening can subsequently be grouped into “risk groups” (e.g., low, population risk, high, and very high) informed by predefined risk thresholds (e.g., high risk for those with more than 8% risk at 40 to 50 years of age; ref. 4). Using this information, it is feasible to offer a risk-stratified NBSP (stratified-NBSP) that adjusts the time between screening intervals to account for the estimated risk of developing a tumor causing breast cancer (medRxiv 2022.12.05.22283099; refs. 5, 6, 7). Women in a very high– or high-risk group would be invited to have a mammogram more frequently than women in the population-risk or low-risk group of developing breast cancer (8). The motivation for implementing a stratified-NBSP is to improve the ratio of health benefits, harms, and healthcare costs, which are the criteria used by the UK National Screening Committee to decide whether to introduce a new screening program or adapt existing screening programs (5).

The cost of implementing a risk-stratified approach within an NBSP in the United Kingdom has been estimated to be £6.64 per woman, generating a total UK population cost of £1,460,800 per year (at 2021 prices; ref. 6). There are a number of published cost-effectiveness analyses using decision analytic models, indicating that a stratified-NBSP could (in principle) offer a better use of the available healthcare budget for providing breast screening in the UK setting (medRxiv 2022.12.05.22283099; refs. 7, 9, 10). Each of these analyses uses a different decision analytic model, underpinning different assumptions, model structures, and data. In 2017, Gray and colleagues published an early-decision analytic model to explore the possibility of introducing a stratified-NBSP. This model was subsequently updated (in 2022) and validated to generate an open access version (MANC-RISK-SCREEN; medRxiv 2022.12.05.22283099). In 2018, the analysis published by Pashayan and colleagues focused on the effect of reducing the time interval between screening for women not at high risk of breast cancer while varying thresholds used to define the “high-risk” category (10). Subsequently, in 2023, Hill and colleagues published an analysis underpinned using a different decision analytic model, which omitted an explicit tumor growth component, but produced consistent results indicating that strategies offering more screening for women at high risk and reduced screening for those at lower risk were likely to be cost-effective in the United Kingdom (9). Hill and colleagues (11) further built on this work by evaluating the cost-effectiveness of an artificial intelligence (AI)–based stratification process in 2024.

A key limitation of the published cost-effectiveness analyses of stratified-NBSP is that they assumed perfect prediction of individual women’s risks and the subsequent assignment to low-, population-, high- or very high–risk groups of developing breast cancer. Despite this assumption, the risk predictions generated by the Tyrer–Cuzick questionnaire are known to be imperfect; a 2018 study by Brentnall and colleagues (12) followed up women assigned to risk groups after 10 years, compared the number of observed breast cancer cases with the number of expected cases in each group, and found fewer cases than expected among women younger than 60 years. The present study aimed to identify the effect of implementing an exemplar stratified-NBSP compared with the current NBSP, 2-yearly screening, or no screening on healthcare costs and health consequences, taking into account the uncertainty in prediction and assignment of risk.

## Materials and Methods

This analysis used a modified version of a published individual-level discrete event simulation model (MANC-RISK-SCREEN; RRID: SCR_028445). This study followed published criteria for reporting model-based cost-effectiveness analyses with the Consolidated Health Economic Evaluation Reporting Standards checklist included in Supplementary Table S1 (13). Table 1 describes the research question and design criteria for the analysis.

**Table 1.** Research questions and key study design criteria.

Decision problem	What are the incremental costs and health consequences of exemplar stratified-NBSP compared with the current NBSP?
Interventions	Three exemplar stratified-NBSPs
	Risk 1: a stratified-NBSP using 10-year risk of breast cancer predicted using the Tyrer–Cuzick questionnaire and Volpara automated breast density measurement (14). Three risk strata (with associated screening intervals) were defined by 10-year risks of breast cancer of (i) <5% (3 yearly); (ii) 5% to 8% (2 yearly); and (iii) >8% (annual)
	Risk 2: a stratified-NBSP using 10-year risk of breast cancer generated with the Tyrer–Cuzick questionnaire and Volpara automated breast density measurement but with strata defined by dividing the population into thirds based on risk (tertiles): (i) the lowest risk tertile (3 yearly); (ii) the middle tertile (2 yearly); and (iii) the highest risk tertile (annual)
	Risk 3: a stratified-NBSP using 10-year risk of breast cancer predicted generated with the Tyrer–Cuzick questionnaire and Volpara automated breast density measurement but with women at low risk (<1.5% 10-year risk) assigned to 5-yearly screening intervals
Comparators	Current UK-NBSP: women between 50 and 70 years with screening every 3 years using mammography
	2-yearly national screening program: women between 50 and 70 years with screening every 2 years using mammography
	No screening: no use of mammography in the population for screening purposes. All cancers would present with clinical signs or symptoms
Model type	Validated discrete event simulation^a^ programmed in R (RRID: SCR_001905)
Population	A cohort of women starting at 38 years who survive until at least 50 years and have not previously been diagnosed with cancer
Setting and perspective	National (UK) healthcare service^b^
Time horizon	Lifetime
Costs	National currency (£) at 2024 prices
	Costs inflated using the NHS Cost Inflation Index or Hospital & Community Health Services index (15)
Health consequences	Life-years and QALYs
Discounting	3.5% for both costs and consequences
	Assuming no difference in costs and health consequences for each strategy for women younger than 50 years; only costs, life-years, and QALYs experienced after 50 years were counted
	Discounting started at 50 years in the analysis
Cost-effectiveness threshold	National Institute for Health and Care Excellence–recommended threshold of £20,000 per QALY gained
Output	NHB assuming a threshold of £20,000 per QALY gained

NOTE: The key design criteria chosen for the economic evaluation are listed in the table.

a An updated version of the Gray model (7) subsequently validated (medRxiv 2022.12.05.22283099).

b Costs to individual women were excluded from the analysis.

### Compliance with ethical standards

This study did not collect primary data, and ethical approval is not relevant.

### Interventions

The interventions considered in this analysis are described in Table 1. We considered 3 risk-stratified approaches to breast cancer screening; see Table 1 for details, including the thresholds used to define risk groups. In the first strategy (“risk 1”), women estimated to have a high risk of breast cancer are invited to annual screening, those with a moderate risk to 2-yearly screening, and all other women to 3-yearly screening. Of the stratified NBSPs under consideration in this study, this strategy aligns most closely with current guidelines produced by the National Institute for Health and Care Excellence (NICE) in the United Kingdom for offering additional screening and prevention to women at higher risk of breast cancer (4). Strategy risk 2 divides women into tertiles based on their 10-year risk, with those in the highest third attending annual screening, those in the middle third attending 2-yearly screening, and those in the bottom third attending 3-yearly screening. Risk 3 is identical to risk 1 but with women predicted to have low breast cancer risk invited to less frequent (5-yearly) screening. In all strategies, risk was predicted at the first mammogram attended by a woman in the simulation and was not re-predicted at subsequent screens.

### Comparators

We compared the 3 stratified-NBSP approaches (hereafter risk 1, risk 2, and risk 3) with the current UK-NBSP and no screening (definitions shown in Table 1); the latter provides a mechanism for checking the logical consistency of the results. An additional comparator was added representing an unstratified NBSP that uses 2-yearly screening for all women, which is the approach used in many European countries such as the Netherlands (16). This approach was included to provide evidence about whether it is more intensive screening or risk prediction specifically that offers increased benefits.

### The relevant population

The relevant population for this decision problem (see Table 1) was defined as women, ages 50 years, followed until death or 100 years of age. Women were simulated from 38 years of age to allow time for tumors to develop before the screening program begins at 50 years of age. The size of the population required for a stable ranking of strategies was determined to be 3 million women following a process of repeated analysis of samples of increasing size until stable rankings of net benefit were achieved. Women who develop cancer before 50 years, or who die from other causes before 50 years, were removed from the sample because there would be no difference in the incremental costs and benefits for these women when using different screening strategies. This approach was considered for analytic reasons because it significantly reduces the underlying variation caused by the simulation model.

### The decision analytic model structure

The decision analytic model simulated a sample of 3 million individual women progressing through the chosen screening strategy using a discrete-event simulation built around 4 components: the stratification process, cancer screening, cancer diagnosis and treatment by cancer stage, and death. In addition, the model employs an algebraic approach to calculate the natural history of cancer progression following the identification of a tumor. The component parts of MANC-RISK-SCREEN are described in Supplementary Materials and Methods S1. For a detailed description of the model structure and diagrams illustrating MANC-RISK-SCREEN, see the documentation folder in the GitHub repository (https://github.com/stuwrighthealthecon/MANC-RISK-SCREEN).

#### Natural history of cancer

The natural history of cancer was represented using a mathematical algorithm (see the model diagrams and tumor growth model functional form choice documents in the GitHub repository: https://github.com/stuwrighthealthecon/MANC-RISK-SCREEN). A random number was drawn and compared with the woman’s lifetime cancer risk to determine if she would experience a cancer in her lifetime. If cancer occurred, then the age and size of the cancer at clinical diagnosis were drawn from a distribution. Observed distributions of age at diagnosis are by definition conditional on individuals surviving long enough to receive a diagnosis. The expected age at death due to all-cause mortality among people diagnosed with cancer is therefore higher than that among the general population. To account for this, we applied a correction based on Bayes rule to the distribution of age at diagnosis to ensure that age at death from all-cause mortality and age at diagnosis could be drawn independently while still reflecting observed distributions (technical details are given in the documentation in the GitHub repository: https://github.com/stuwrighthealthecon/MANC-RISK-SCREEN). A tumor growth rate was drawn, and the age of tumorigenesis was back-calculated using the tumor growth model (17). The distribution of tumor growth rates was estimated from data including both invasive cancers and ductal carcinoma *in situ* (DCIS). Over time, the cancer grew at a rate determined by the cancer growth rate and was potentially detectable by screening, conditional on the size of the cancer and whether the woman attended a screen.

#### The stratification process

Each individual woman in the simulation was initially assigned a Volpara breast density score, 10-year risk, and lifetime breast cancer risk based on data from a published study, BC-Predict, in which risk data were calculated for 15,613 women using updated versions of the Tyrer–Cuzick questionnaire and Volpara breast density measurement (14, 18).

#### Cancer screening and clinical detection

Based on the estimated 10-year risk for each woman and the screening strategy implemented, each individual woman was assigned to a prespecified screening interval. At a screening event, the size of the tumor (if present) was determined using the tumor growth model. The sensitivity of screening was assumed to be conditional on the size of the tumor and the woman’s breast density. A random number was drawn against this sensitivity to determine if the tumor was detected.

#### Diagnosis and treatment

When a tumor was identified by screening or when it reached the size at which it would be clinically diagnosed, a cancer stage was assigned based on the distribution of cancers observed at different stages. This includes allocation to DCIS for small cancers. MANC-RISK-SCREEN used tumor–node–metastasis (TNM; extent of the tumor; extent of spread to the lymph nodes; and presence of metastasis) stages. The TNM stage was used rather than other systems, such as the Nottingham Prognostic Indicator (NPI), because more data are available for this grading system (19).

#### Mortality

When a cancer was identified by screening and then diagnosed and allocated a defined stage of disease, a new age of death was drawn to reflect the impact of the stage of cancer on mortality. In MANC-RISK-SCREEN, exponential survival functions were fitted to 5-year survival for tumors of different stages (20). For women diagnosed with stage IV cancers, age also affected survival, with lower survival at higher ages. It was assumed that there was no mortality impact of being diagnosed with DCIS.

### Decision analytic model inputs

There were 3 categories of decision analytic model inputs required for this analysis: clinical input parameters, utility input parameters, and resource use and cost input parameters (see Table 2). The relevant clinical input parameters are cancer incidence accounting for estimated future risk of breast cancer, tumor growth, estimate of 10-year risk, uptake of screening, mammography recall rate, sensitivity and specificity of diagnosis, and success of treatment by cancer stage. Each of these parameters is described in Supplementary Materials and Methods S2 with full details of how each parameter was calculated included in Supplementary Tables S2, S3, and S4.

**Table 2.** Model parameters and sources.

Parameter	Value	Sources and assumptions
Clinical parameters		
Probability of attending a screening appointment	62.5% for the first screen	NHS Digital (21)
	88.7% if 1 previous screen attended	
	20.8% if first screen not attended	
Age of death due to all-cause mortality	∼Weibull (9.38, 89.35)	Office for National Statistics (22)
Survival for stage I breast cancer	∼exp (−5.462)	NHS Digital (23)
Survival for stage II breast cancer	∼exp (−4.023)	NHS Digital (23)
Survival for stage III breast cancer	∼exp (−2.465)	NHS Digital (23)
Survival for stage IV breast cancer for women up to 54 years	∼exp (−1.787)	Office for National Statistics (20)
Survival for stage IV breast cancer for women 55 to 74 years	∼exp (−1.388)	Office for National Statistics (20)
Survival for stage IV breast cancer for women older than 75 years	∼exp (−1.011)	Office for National Statistics (20)
Breast cancer incidence by age	See Supplementary Table S5	Cancer Research UK (24)
10-year risk of breast cancer estimated using the Tyrer–Cuzick version 8 and Volpara breast density group	See Supplementary Table S5	Observations of breast density (using Volpara TruDensity), 10-year breast cancer risk (using Tyrer–Cuzick version 8 and Volpara TruDensity), and lifetime risk (using Tyrer–Cuzick version 8 and Volpara TruDensity) for 15,613 women were shared securely by the BC-Predict research team^a^
Lifetime risk of breast cancer estimated using the Tyrer–Cuzick version 8 and Volpara breast density group	See Supplementary Table S5	Observations of breast density (using Volpara TruDensity), 10-year breast cancer risk (using Tyrer–Cuzick version 8 and Volpara TruDensity), and lifetime risk (using Tyrer–Cuzick version 8 and Volpara TruDensity) for 15,613 women were shared securely by the BC-Predict research team^a^
Volpara breast density estimate	See Supplementary Table S5	Observations of breast density (using Volpara TruDensity), 10-year breast cancer risk (using Tyrer–Cuzick version 8 and Volpara TruDensity), and lifetime risk (using Tyrer–Cuzick version 8 and Volpara TruDensity) for 15,613 women were shared securely by the BC-Predict research team^a^
Probability that a cancer is metastatic given the age of the woman	25 years = 4.62%	Age nearest to woman’s current age at diagnosis of cancer used
	35 years = 8.67%	
	45 years = 10.98%	
	55 years = 12.71%	
	65 years = 14.25%	
	75 years = 15.98%	
	85 years = 17.30%	
Probability of cancer being diagnosed as stage I, II, or III given the size of tumor	See Supplementary Table S6	Kollias and colleagues (25) Wen and colleagues (26) Cheng and colleagues (27)
Mammographic sensitivity by VDG	VDG1 = 75%	Payne and colleagues (28)
	VDG2 = 73.5%%	
	VDG3 = 59.8%	
	VDG4 = 51.3%	
	Average = 66.6%	
Recall (false-positive) rate for screening	3.1%	NHS Digital (21)
Biopsy rate for recalls	50.6%	NHS Digital (21)
Growth model parameters		
Mean tumor doublings at clinical detection	6.5	Weedon-Fekjaer and colleagues (17)
Standard deviation of tumor doublings at clinical detection	0.535	Weedon-Fekjaeær and colleagues (17)
Log normal mean of tumor growth rate	1.07	Weedon-Fekjaer and colleagues (17)
Log normal standard deviation of tumor growth rate	1.31	Weedon-Fekjaer and colleagues (17)
Maximum tumor size (mm)	128	Weedon-Fekjaer and colleagues (17)
Starting tumor size (mm)	0.25	Weedon-Fekjaer and colleagues (17)
Utility parameters	​	​
Age-related utility values for healthy individuals	See Supplementary Table S7	Hernandez Alava and colleagues (29)
Utility early breast cancer in the first year	0.82	Naik and colleagues (30)
Utility early breast cancer in subsequent years	0.82	Naik and colleagues (30)
Advanced breast cancer in the first year	0.75	Naik and colleagues (30)
Advanced breast cancer in subsequent years	0.75	Naik and colleagues (30)
Cost parameters	​	​
Cost of risk stratification	£7.40	Wright and colleagues (6) updated to 2024 values
Cost of mammography screen	£39.93	Microcosting study See model parameter update document in GitHub (https://github.com/stuwrighthealthecon/MANC-RISK-SCREEN)
Cost of follow-up	£130.97	Gray and colleagues (7) updated to 2024 values
Cost of biopsy	£558	NHS England (31)
Cost of ultrasound screening	£102	NHS England (31)
Cost of MRI screening	£162	NHS England (31)
Cost of treating breast cancer by stage, age, and time lived with cancer	See Supplementary Table S8	Laudicella and colleagues (32)
Cost of treating a DCIS	£12,346.83	Gray and colleagues (7) updated to 2024 values

NOTE: This table lists the clinical, cost, and utility values used as parameter inputs in the model and cites the source of the value or explains how it was calculated.

Abbreviations: exp, exponential; VDG, Volpara Density Group.

a To allow these data to be shared outside the research team, a synthetic data set was created using the synthpop package in R. This approach resulted in a data set with 15,613 synthetically generated observations of breast density, 10 year risk, and lifetime risk. The approach also preserves the structure of the underlying data including correlations.

#### Health consequences

Two relevant health consequences were included in this analysis: life-years (representing life expectancy) and quality-adjusted life-years (QALY). To generate estimates of the life expectancy of a woman without breast cancer, updated national population life tables were used to estimate the parameters for a Weibull survival distribution (33). Life expectancy data were estimated using data collected before the COVID-19 pandemic starting in February 2020. Survival estimates for breast cancer of stages I to III inclusive were taken from the 5-year cancer survival rates estimated by National Health Service (NHS) Digital (23). Due to the limited availability of survival data, only exponential survival functions could be fitted to these estimates, although this was found to be the optimal choice of survival model in the “Gray model” that used survival by NPI grade (7). For stage IV cancers, exponential survival models were estimated in the same manner but for different age bands (38–54 years; 55–74 years; and 75–99 years) to reflect the effects of age on cancer survival.

When a woman was assumed to be diagnosed with cancer, an age at death from cancer was drawn using the survival function for her stage of cancer and age, and the woman’s age at death was set to be the minimum of the age at death from cancer and the age at death from all-cause mortality. For screen-detected cancers, the new mortality age was calculated based on the time the cancer would have been detected clinically. This approach avoided a potential problem in which women with similar-stage cancers detected earlier by screening also died at an earlier age.

Published values for health consequences (utility values) for different stages of breast cancer, relevant to the UK setting, were identified from studies included in 2 recently published systematic reviews of breast cancer–related utility values (34, 35). The face validity of these published utility values was assessed using a group of patient experts (3 women) with experience of breast cancer. In an online meeting, facilitated by 2 researchers, the women were asked to choose which they thought best reflected the experience of women in the United Kingdom with breast cancer. The final chosen utility values were those from Naik and colleagues (30). The women involved in the patient and public involvement (PPI) group suggested that one of the key reasons for choosing the Naik and colleagues (30) utilities was that health-related quality of life does not improve after the first year of diagnoses. Each member of the PPI group highlighted the long-lasting psychologic impact of having previously been diagnosed with breast cancer. The full process for choosing these utility values is detailed in the “parameter update version 0 to 1” documentation file of the model GitHub repository (https://github.com/stuwrighthealthecon/MANC-RISK-SCREEN).

In the model, women were assigned age-related utility values for each year they were alive, reflecting decreasing baselines quality of life as age increases. The analysis assumes that when a woman was diagnosed with cancer, their baseline age-related utility value was multiplied by the stage-related utility value to ensure that the assumed health-related quality of life when assigned to a cancer state was not higher than when the healthy (non-cancer) state was assigned.

#### Resource use and costs

The selected perspective for this study was the UK healthcare system (the NHS). The items of resource use and associated costs for screening, diagnosis, and treatment were included in the analysis.

The cost of predicting a woman’s risk of breast cancer was estimated in a published microcosting study (6). This microcosting study estimated costs for 3 risk prediction strategies assuming a trial setting and in the NHS. This analysis used the estimated cost for the Tyrer–Cuzick questionnaire and Volpara breast density measurement in the NHS, inflated to 2024 prices.

For each screening event, costs associated with each item of resource use were included to reflect the type of technology used. The cost of risk stratification was added at the first screen. Costs are assigned for false-positive results and biopsies when these events occur.

Accurate costs of mammography for breast cancer screening were not available in the United Kingdom. A microcosting study was conducted in consultation with members of the BC-Predict research team. Full details of the calculation of mammography cost are available in the parameter update document included in the GitHub repository (https://github.com/stuwrighthealthecon/MANC-RISK-SCREEN). The cost of recall for false-positive results was inflated from the published 2021 values used in the Gray model. The cost of biopsy was taken from NHS reference costs (36).

When a woman in the model was assumed to be diagnosed with breast cancer, a lookup table was used to assign the total cost of treatment given the stage of cancer, age at diagnosis, and number of years the woman will live with breast cancer (32). As Laudicella and colleagues (32) only estimated the costs of treatment up to 9 years following diagnosis, it was assumed that there were no additional costs past this point even if a woman survived with cancer for this long. This is to prevent overly large costs being assigned in rare instances in which women with lower stage cancers have very long survival times.

A systematic review was used to identify the assumed treatment costs for each stage of breast cancer. Full details of this review can be found in the parameter update document in the documentation folder in the GitHub repository (https://github.com/stuwrighthealthecon/MANC-RISK-SCREEN). Only 1 study (Laudicella and colleagues; ref. 32) which was relevant to the United Kingdom was identified. These data were used to create an exponential regression predicting the total treatment cost for a woman given her age at diagnosis, cancer stage at diagnosis, and life expectancy. The treatment costs were appropriately assigned a discount rate (see Table 1). The cost of treating non-invasive tumors was not included in Laudicella and colleagues, and it was necessary to use the cost assumed in Gray and colleagues and inflate this cost to 2024 values (7, 32).

### Data analysis

The main (base case) analysis calculated the expected healthcare costs, life-years, and QALYs for each strategy. The appropriate discount rate (see Table 1) was assigned to costs, life-years, and QALYs. A fully incremental analysis was conducted. There was no published threshold for cost-effectiveness recommended by the UK National Screening Committee, and so we assumed a threshold of £20,000 per QALY gained in line with that used by NICE (37). The net health benefit (NHB) produced by each strategy was also calculated using a threshold of £20,000 per QALY in order to provide a ranking of the strategies. NHB was calculated as the QALYs produced by the intervention minus the cost of the program divided by the threshold. In addition to costs and QALYs, the average number of screens attended by women in each strategy was also recorded to demonstrate the potential resource impact of different screening strategies.

The (stochastic) simulation model built for this analysis captured 2 levels of uncertainty inherent in the screening population health systems. First-order uncertainty represents the variability in outcomes among the individuals in the model due to variation in their individual characteristics and in their experience of screening, as well as the inherently random nature of cancer incidence. Second-order uncertainty refers to uncertainty in the outcomes due to uncertainty in the true values of the input parameters. In this model, we conduct 2 types of analyses; in the “base-case” analysis, we simulate sufficient numbers of women to flow through the model to generate stable central estimates of costs and QALYs for the cohort. In the probabilistic sensitivity analysis, we explore the impact of uncertainty in the parameter estimates used in the model.

For the base-case analysis, 2 scenarios were explored: perfect risk prediction and error in risk classification. In the first scenario, it was assumed that a woman’s predicted risk of breast cancer represented her true risk. In the second scenario, error in the risk prediction algorithm was accounted for by including each woman’s predicted and corrected risk estimates (see Supplementary Materials and Methods S3). Outcomes are presented and compared for each scenario using tables and cost-effectiveness frontiers.

Our probabilistic sensitivity analysis explored the impact of joint parameter uncertainty on the optimal choice of NBSP. The distributions chosen for the parameters are shown in Supplementary Tables S9–S12. Due to the number of simulations required to produce stable estimates, it was not feasible to run a large number of Monte Carlo simulations for the probabilistic analysis. Instead, Monte Carlo simulations were conducted using wide distributions for the parameter estimates, and generalized additive models (GAM) fitted to predict the costs and QALYs for each strategy given the values of the input parameters. A dataset of 1 million simulations of the parameter values was then generated using the realistic parameter distributions, and the GAM models were used to estimate costs and QALYs for each strategy for each parameter set. The results of this analysis were then used to produce cost-effectiveness acceptability curves. Due to the complexity of conducting a probabilistic sensitivity analysis, the analysis was only conducted for the more likely scenario that there was an error in risk prediction. Additional analysis, reported in Supplementary Figs. S1 and S2, was also conducted to determine the impact of changes in the parameter values on the optimal screening strategy. A scenario analysis was conducted in which the assumed discount rate was changed from 3.5% to 1.5% (see Supplementary Table S13; Supplementary Fig. S3). Although the main focus of this study was the cost-effectiveness of different strategies, the model predictions of a selection of clinical outcomes under different risk-based approaches to breast screening were also evaluated in a supplementary analysis (see Supplementary Table S14).

## Results

### Cost-effectiveness assuming perfect risk prediction

**Figure 1.**
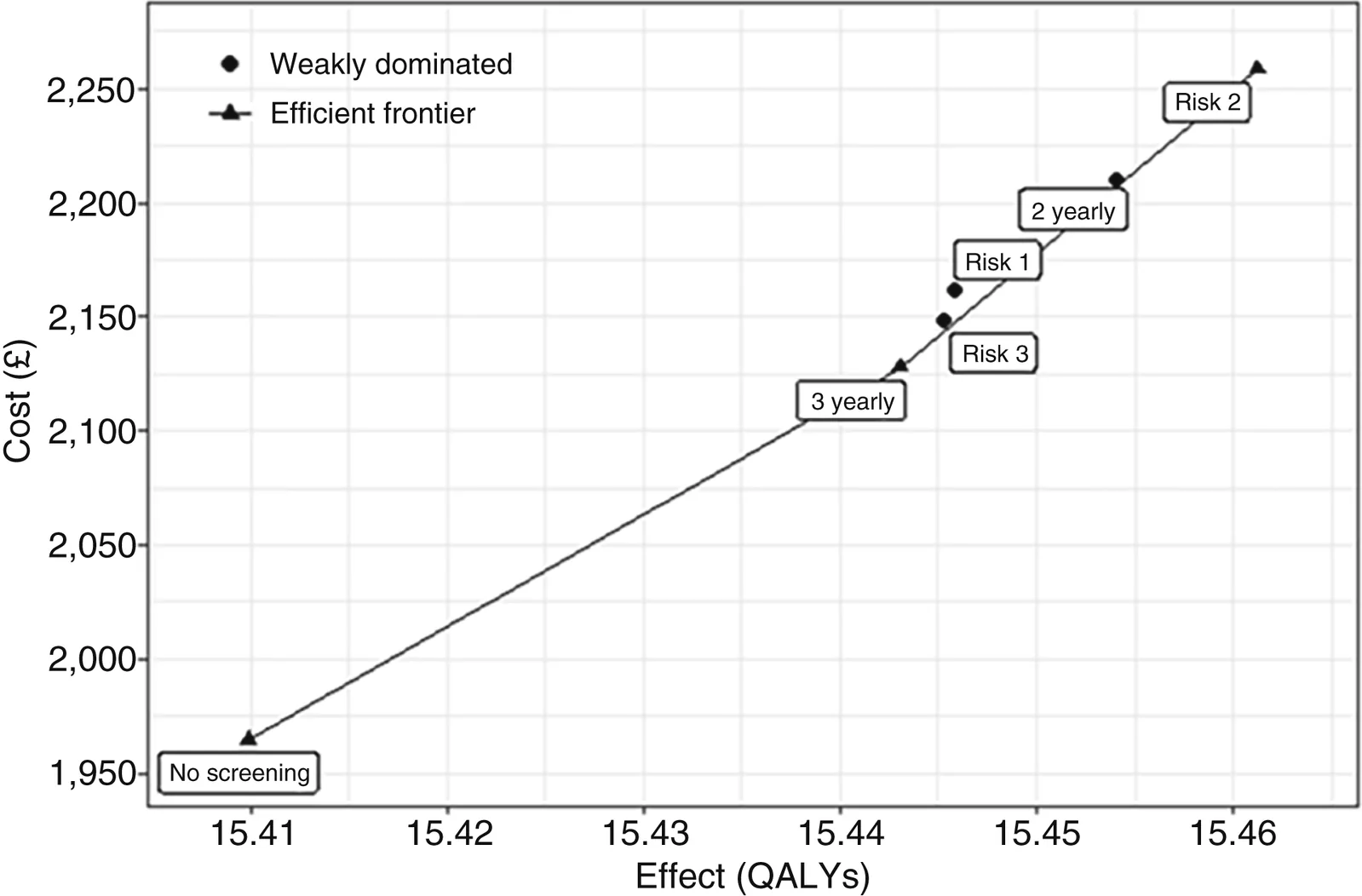
Cost-effectiveness frontier when risk prediction is assumed to be perfect. Incremental QALYs and costs resulting from the different strategies modeled in the economic evaluation when risk prediction was assumed to be perfect. Strategies on the frontier shown are potentially cost-effective.

The strategy which provided the greatest NHB when assuming perfect risk prediction was risk 2 (representing a strategy that split the sample into risk tertiles). The strategies which provided the next greatest benefit in terms of NHB were 2-yearly screening, risk 3, and risk 1. The cost-effectiveness plane displaying the results assuming perfect risk prediction is presented in Fig. 1, and the ranking of strategies is presented in Table 3.

**Table 3.** Base-case results of MANC-RISK-SCREEN^a^.

Strategy	Cost (£)^b^	Effect (QALYs)^a^	Screen	Incremental cost (£)	Incremental effect (QALYs)	Incremental cost-effectiveness ratio	NHB^c^	Ranking health benefit
Cost-effectiveness results assuming perfect risk prediction								
No screening	£1,965	15.410	0	—	—	—	15.3117	6
3 yearly	£2,128	15.443	4.65	£163	0.033	£4,914 per QALY	15.3367	5
Risk 3	£2,149	15.445	4.82	£21	0.002	Extendedly dominated	15.3378	3
Risk 1	£2,162	15.446	5.07	£34	0.003	Extendedly dominated	15.3377	4
2 yearly	£2,210	15.454	7.68	£82	0.011	Extendedly dominated	15.3436	2
Risk 2	£2,258	15.462	9.28	£131	0.018	£7,200 per QALY	15.3483	1
Cost-effectiveness results accounting for risk misclassification								
No screening	£1,547	15.468	0	—	—	—	15.39016	6
3 yearly	£1,834	15.492	4.70	287	0.02485	£11,543 per QALY	15.40067	4
Risk 3	£1,854	15.493	4.89	21	0.00109	Extendedly dominated	15.40072	3
Risk 1	£1,865	15.495	5.14	31	0.00255	£12,438 per QALY	15.40163	1
2 yearly	£1,998	15.501	7.77	133	0.00595	£22,325 per QALY	15.40094	2
Risk 2	£2,091	15.505	9.43	93	0.00372	£24,946 per QALY	15.40002	5

NOTE: This table lists the core results of the economic evaluation including the costs, QALYs, average number of screens, incremental cost-effectiveness ratio, and the ranking of the strategies in terms of net benefit.

a Risk 1: a stratified-NBSP using 10-year risk of breast cancer predicted using the Tyrer–Cuzick questionnaire and Volpara automated breast density measurement. Risk 2: a stratified-NBSP using 10-year risk of breast cancer generated with the Tyrer–Cuzick questionnaire and Volpara automated breast density measurement but with strata defined by dividing the population into thirds based on risk (tertiles). Risk 3: a stratified-NBSP using 10-year risk of breast cancer predicted generated with the Tyrer–Cuzick questionnaire and Volpara automated breast density measurement but with women at low risk (<1.5% 10 year risk) assigned to 5-yearly screening intervals. 3 yearly: women between 50 and 70 years with screening every 3 years using mammography. 2 yearly: women between 50 and 70 years with screening every 2 years using mammography. No screening: no use of mammography in the population for screening purposes.

b All reported costs and effects discounted at a rate of 3.5%.

c At a threshold of £20,000 per QALY.

Although risk 2 was the most cost-effective strategy (in terms of largest NHB), it was also very resource intensive, requiring women to attend an average of 9.28 screens—nearly double the number of screening events expected in current practice. Conversely, risk 1 and risk 3 required women to attend an average of 5.07 and 4.82 screens, respectively, representing an increase of only 0.42 and 0.17 screens compared with 3-yearly screening (4.65).

### Cost-effectiveness accounting for risk misclassification

**Figure 2.**
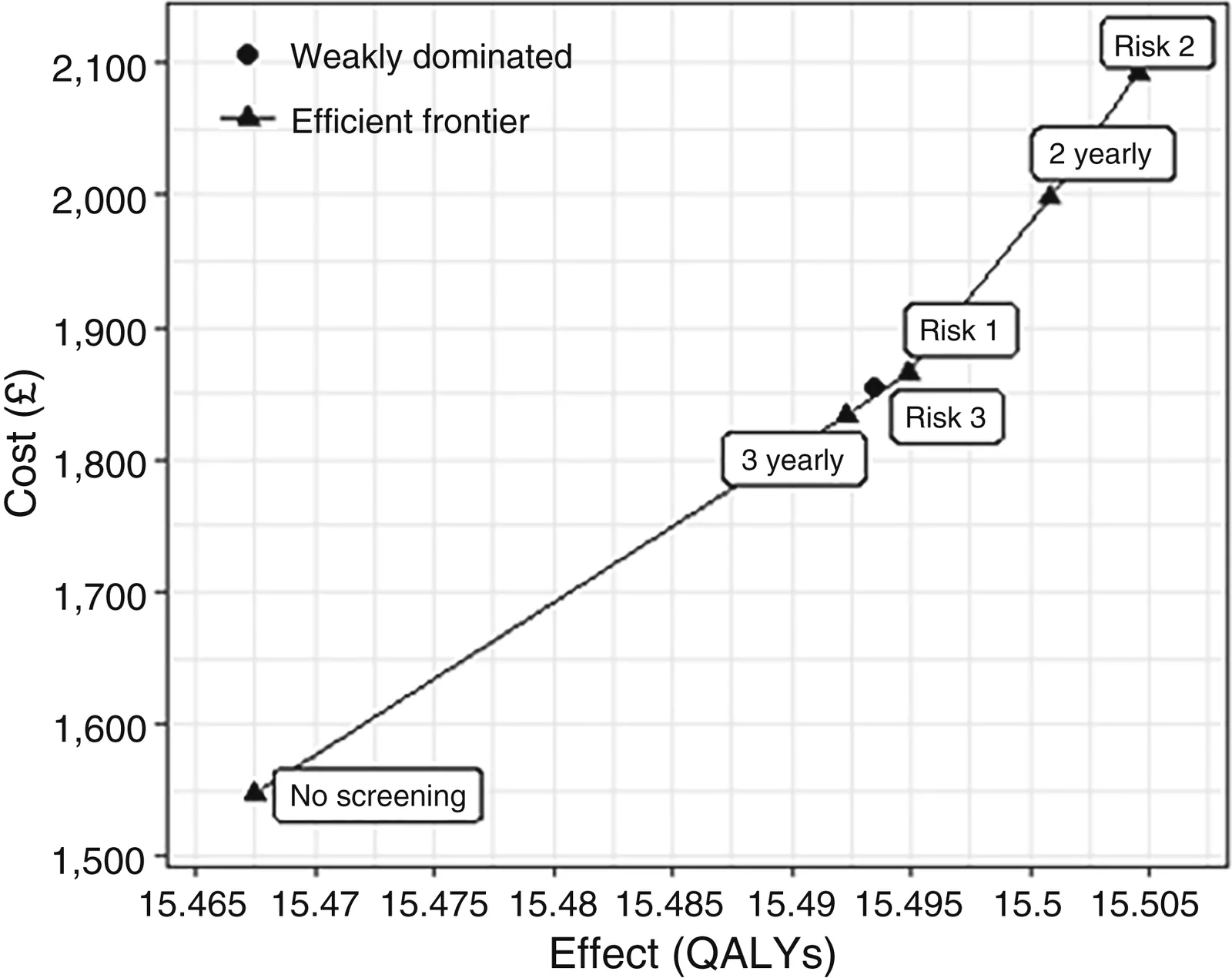
Cost-effectiveness frontier accounting for risk misclassification. Incremental QALYs and costs resulting from the different strategies modeled in the economic evaluation when error in risk prediction was accounted for. Strategies on the frontier shown are potentially cost-effective.

When accounting for the potential for the risk prediction algorithm to misclassify women into the allocated risk group, the calculated NHB changed. The risk 1 strategy was now found to produce the highest NHB of the strategies. This was followed by 2-yearly screening and risk 3. Risk 2, which had been found to be the most cost-effective approach when assuming perfect prediction, had the second lowest NHB. The cost-effectiveness plane presenting the results of the analysis when assuming imperfect risk prediction are shown in Fig. 2, and the ranking of NHB is shown in Table 3.

When accounting for misclassification, the total number of screens for each strategy increased marginally. The average number of screens increased by a small amount for universal strategies (0.05 for 3 yearly and 0.09 for 2 yearly), as with fewer cancers in the population, more people lived cancer-free for longer and therefore underwent more screens. For the risk-based approach, the average number of screens also increased, with the largest increase observed for the most intense strategy, risk 2 (0.15 screens). Smaller increases were observed for risk 1 (0.07) and risk 3 (0.07).

### Probabilistic results

**Figure 3.**
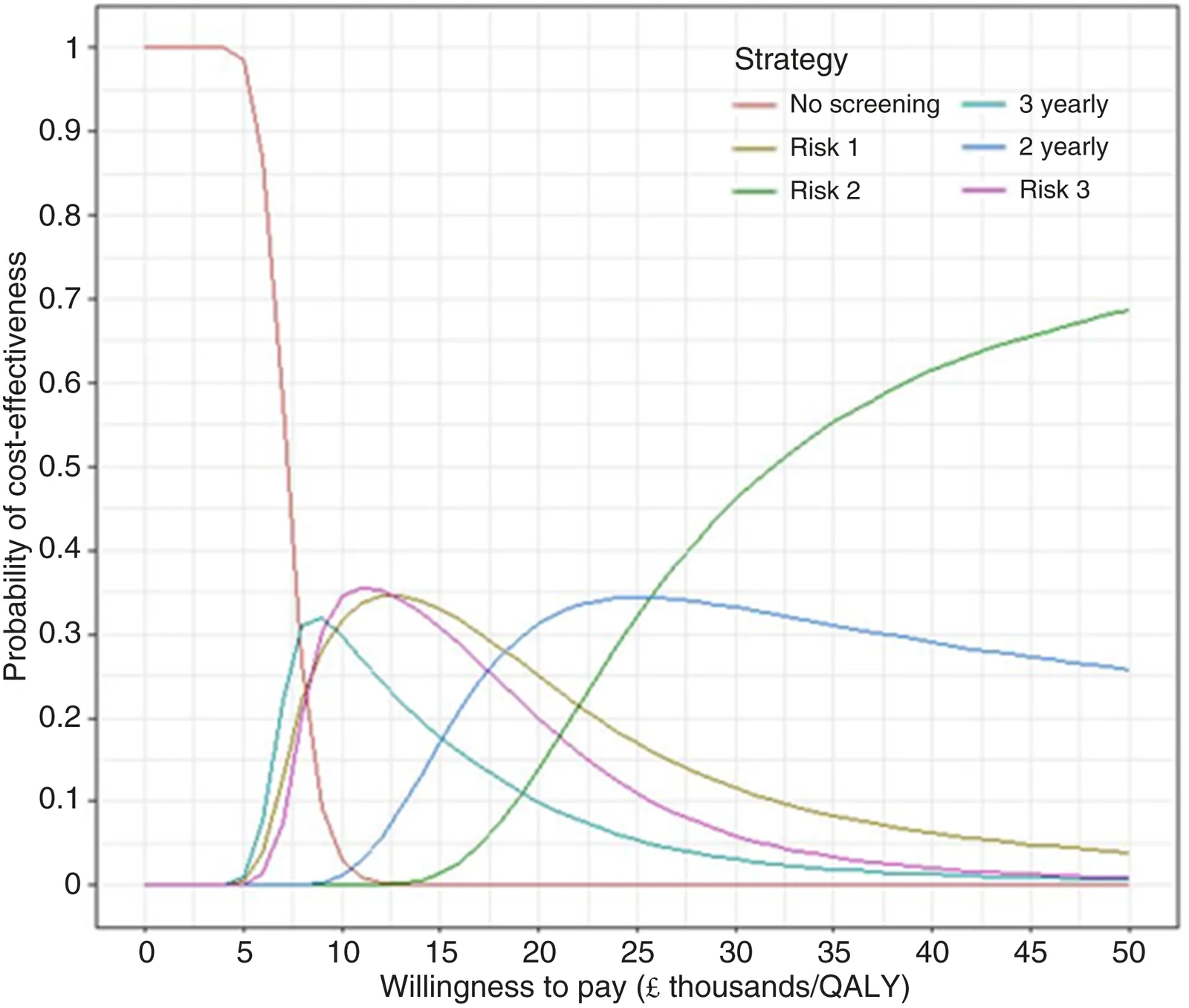
Cost-effectiveness acceptability curve. Probability of each strategy being the most cost-effective at varying cost-effectiveness thresholds.

The cost-effectiveness acceptability curve in Fig. 3 illustrates the impact of assuming different thresholds for cost-effectiveness. At thresholds lower than £7,500 per QALY, no screening is the strategy most likely to be cost-effective. At this threshold, 3-yearly screening, risk 1, and risk 3 show similar probabilities of being most cost-effective. Risk 1 is most likely to be cost-effective between thresholds of around £12,500 per QALY and £18,00 per QALY, followed by 2-yearly screening, which becomes the most likely to be cost-effective. At thresholds higher than £25,000, the risk 2 strategy becomes the most cost-effective. There is a consistent pattern in the results, as the cost-effectiveness threshold increases, more screen-intensive strategies become more likely to be the most cost-effective option.

### Additional analyses

Additional analyses (see Supplementary Figs. S1 and S2) show the impact of changes in parameter values on the strategy generating the highest NHB at a threshold of £20,000 per QALY. Although the risk 1 strategy was likely to generate the highest NHB in most scenarios, the 2-yearly screening strategy was more likely to generate the highest NHB when survival for cancer stages I to III, health utility values, or screening-related costs were lower than assumed in the base-case analysis.

The results of the analysis obtained by reducing the discount rate to 1.5% for both health outcomes and costs are shown in Supplementary Table S13; Supplementary Fig. S3. In this scenario, the risk 2 strategy generated the highest NHB at thresholds of both £20,000 and £30,000 per QALY.

The illustrative clinical predictions of the model under different risk-stratified screening scenarios are presented in Supplementary Table S14. For simplicity, the results of 2-yearly screening and risk 1 are discussed here in comparison with 3-yearly screening, as these were most likely to be cost-effective at a threshold of £20,000 per QALY. Risk 1 and 2-yearly screening strategies increase the detection of cancers through screening from 22.55 per 1,000 women invited to 24.75 and 30.28 per 1,000 women invited, respectively. However, this increased screening intensity was also reflected in increased lifetime false-positive rates of 179.99 per 1,000 woman invited (for risk 1) and 272.04 per 1,000 women (2 yearly) compared with 164.52 per 1,000 women in 3-yearly screening. The mean cancer diameter at screen detection decreased from 12 mm (3 yearly) to 11.7 mm (risk 1) and 11.3 mm (2 yearly). Average cancer-specific survival for women diagnosed with cancer after 50 years of age increased by 54.75 days in the risk 1 strategy and 120.45 days in 2-yearly screening.

## Discussion

This study reported a decision analytic model–based cost-effectiveness analysis of implementing a stratified-NBSP. The assumed risk prediction approach was published in BC-Predict (14, 38). Accounting for error in breast cancer risk misclassification arising from the risk prediction algorithm generated a different ranking of strategies compared with the ranking obtained assuming perfect prediction; however, our results suggest that risk-based screening strategies will produce a higher NHB than universal breast cancer screening strategies under either set of assumptions.

At cost-effectiveness thresholds between £11,000 per QALY and £16,000 per QALY, a risk-stratified breast cancer screening approach was better than universal screening. Between thresholds of £16,000 per QALY and £25,000 per QALY, 2-yearly universal screening was the approach most likely to be cost-effective. In addition, stratified screening involving more intensive screening was more cost-effective than less intensive screening at thresholds over £25,000 per QALY.

Many countries, such as the United States and European nations such as the Netherlands, currently offer 2-yearly screening. This study suggests that such an approach would be cost-effective in the United Kingdom compared with current screening, although capacity issues may mean that it is not feasible in practice. Further research with models adapted to country-specific contexts is needed to determine if risk-stratified screening offers potential benefits in better targeting screening in countries with existing intensive screening strategies.

Given the UK cost-effectiveness threshold of £20,000 per QALY and the existence of capacity constraints in the system such as the number of available radiographers and radiologists and in turn the number of mammograms performed, a risk-stratified approach which enhances cancer detection while minimizing the number of additional screens is likely to be an attractive strategy in the NHS. Reducing the number of screens for women at lower risk of cancer may save resources, but the cost-effectiveness of such a strategy is at best equivalent to offering 3-yearly screening to lower-risk women. Given the potentially infinite number of combinations of risk prediction approach, risk thresholds, and screening intervals, further research is needed to identify the strategy which maximizes net benefit for a constrained number of screens.

Alongside the potential introduction of risk-stratified breast screening, there are a number of potentially promising interventions which may change the way breast screening is implemented in health systems. Studies such as the Breast screening—Risk Adapted Imaging for Density (BRAID) trial have shown that supplemental screening with different imaging modalities may aid in the detection of cancers in women with dense breasts (39). Furthermore, the introduction of AI-based mammography reading may potentially reduce workloads for radiologists if studies suggest that such models show similar sensitivity and specificity (40). Each of these interventions also interacts with the others; for example, AI algorithms could be used to read an initial mammogram, determine breast density, and stratify women to different screening modalities or intervals based on the results. Further modeling will be critical to determine the optimal combination of these strategies to inform decision-makers about how best to implement them within the health system.

The results of this study support findings by Hill and colleagues (9) that risk-based screening approaches are likely to be superior to 3-yearly screening in the UK-NBSP. It is difficult to directly compare the results of this study with those of Hill and colleagues as some of the strategies in the two models are similar. The MANC-RISK-SCREEN model uses updated risk thresholds for the moderate-risk group (5%–8%), whereas the Hill model uses the thresholds used in the Gray model (3.5%–8%). For future evaluations of risk-stratified breast cancer screening in the United Kingdom, it would be useful to use MANC-RISK-SCREEN, Hill and colleagues, and Pashayan and colleagues models to evaluate prespecified stratified screening strategies in order to directly compare the results of the models (9, 10).

### Limitations

The validation of MANC-RISK-SCREEN suggested some inherent weaknesses in the ability to produce outputs that match those observed in reality (medRxiv 2022.12.05.22283099). Although the distribution of the tumor stage identified at screening was similar between the model predictions and observed values, across all cancers the model tended to generate too many cancers at stage III and too few at stage I.

This modeling study is underpinned by a breast cancer growth model, allowing for the impact of early detection on cancer size and resulting stage to be modeled. Because it is difficult to estimate natural history models when screening is implemented in the system, this growth model was estimated using data from Norwegian women collected before and after the Norwegian program was introduced. This population may not be representative of the UK population at present, particularly with regard to ethnicity which may be linked to cancer risk and cancer growth. In addition, other correlations between factors, such as socioeconomic status, which influence both attendance at screening and the risk of cancer, have not been included in this analysis. Although the predictions of cancer size at screening and clinical detection align closely with estimates from studies of tumor doubling time in the United Kingdom, further research is needed to explore differences in outcomes by ethnicity and socioeconomic status and the potential for risk-stratified screening to compound existing inequalities in breast screening (41, 42).

The analysis using MANC-RISK-SCREEN has suggested that using the Tyrer–Cuzick questionnaire and Volpara breast density estimates to predict risk may be a cost-effective use of resources which does not materially increase the number of mammography scans across the NHS. However, there are many approaches to risk prediction that could be combined in a large number of ways to predict a woman’s risk. For example, the CanRisk approach has been developed by researchers at the University of Cambridge, building on the BOADICEA risk prediction model (43). Further research is needed to understand the most cost-effective approach to risk prediction for risk-stratified breast cancer screening. This should involve a full incremental analysis such that the costs and health benefits for the different risk prediction approaches are compared against all other available approaches as opposed to only 3-yearly screening.

This study assumes that if risk-stratified breast screening were introduced into the UK-NBSP, it would be implemented perfectly. It was assumed that all women in the program would choose to have their risk predicted and would agree to change their screening intervals. In reality, qualitative research has suggested that women may be reluctant to reduce their breast cancer screening interval when predicted to be at low risk. In addition, there may be barriers to introducing a risk prediction questionnaire and automated breast density measurement into the screening program. For example, in the BC-Predict trial, issues were faced in ensuring accuracy of the risk information entered into the system and in transmitting images for breast density calculation. Wright and colleagues (6) previously estimated that such barriers could increase the cost per person of offering risk prediction from £8.46 per woman to £20.56 (£; at 2021 prices). This could affect the cost-effectiveness of risk-stratified screening in practice.

In published economic evaluations, the cost of mammography screening has been taken from NHS reference costs. However, in recent years, the cost of mammography for screening has not been included in the reference costs, and simply inflating the last available cost may provide inaccurate estimates. In this study, we used the results of a microcosting exercise undertaken through communication with experienced researchers in the screening program.

Updated treatment costs for different stages of cancer are also required for future versions of the model. The data included in this analysis relate to resource use from 2001 to 2010. Since these dates, treatment pathways for breast cancer will have changed significantly, resulting in changes in the pattern of costs by cancer stage. The cost of many of these treatments, evaluated by NICE, is confidential, making it difficult to discern their true impact on total treatment costs. In addition, Laudicella and colleagues (32) only break down costs into early (stage I and II) and late (III and IV) cancers, limiting the granularity of data and only providing a significant benefit of screening when there is a stage shift from stage III to II. In addition, the costs of treating DCIS were not included in this article, and the values included in this study are even more out of date.

Future modeling of the cost-effectiveness of risk-stratified screening should seek to add greater granularity to the parameters underpinning the analysis. For example, rather than using a single false-positive rate, this should be varied by risk group in strategies and varying false-positive rates in different age groups and breast densities should be considered. The MANC-RISK-SCREEN model is currently being further developed in projects to evaluate the cost-effectiveness of supplemental screening for women with dense breasts and the introduction of AI-based mammogram reading. In these studies, further detail will be added to the model. This will also facilitate the modeling of strategies combining elements of risk stratification, supplemental screening, and AI.

Due to negligible differences in estimated QALYs between the strategies, a large number of simulated women are required to produce accurate estimates in the analysis. Stability was achieved in the results for the base-case analysis, but there is less certainty with regard to stability of the results of the probabilistic analysis, resulting in the use of the GAM model. This is particularly important when comparing the cost-effectiveness of risk 1 and risk 3, which only differ in the reduction of screening for women with a 10-year cancer risk of less than 1.5%.

### Conclusions

This cost-effectiveness analysis suggests that risk-stratified breast cancer screening is likely to be cost-effective when compared with universal breast screening strategies. Error in risk prediction may result in the misclassification of women into risk groups. Although this has a minimal impact on the cost-effectiveness of risk-stratified screening, it may affect the choice of the most cost-effective approach in practice.

## Supplementary Material

Supplementary Methods A file containing additional details about the methods used in the article

Supplementary Figure S1 The impact of changes to the parameter values on the most cost-effective strategy at a thresholds of £20,000 per QALY

Supplementary Figure S2 The impact of changes to the parameter values on the most cost-effective strategy at a thresholds of £20,000 per QALY

Supplementary Figure S3 A graph showing the additional costs and QALYs for each strategy when a discount rate of 1.5% is used instead of 3.5%

Supplementary Table S1 CHEERS Checklist

Supplementary Table S2 Clinical parameters in deterministic model

Supplementary Table S3 Costs used in deterministic model

Supplementary Table S4 Utility values used in the deterministic model

Supplementary Table S5 A table containing observations of breast density, 10 yr risk and lifetime risk

Supplementary Table S6 A table containing the probability of cancer being metastatic by age

Supplementary Table S7 A table containing utility values by age

Supplementary Table S8 A table showing the cost of treating cancers by stage and age

Supplementary Table S9 Distributions used in the probablistic analysis

Supplementary Table S10 Covariance matrix for survival post-breast cancer

Supplementary Table S11 Covariance matrix for metastatic survival

Supplementary Table S12 Covariance matrix for utility values

Supplementary Table S13 Results when using a discount rate of 1.5%

Supplementary Table S14 Predicted Clinical Outcomes Under Different Screening Strategies

## Data Availability

This study did not collect primary data. The model code and data used to inform the model are available on GitHub: https://github.com/stuwrighthealthecon/MANC-RISK-SCREEN. Supplementary model documentation can be found in Model Core Files/Documentation.

## Code Availability

The model code can also be downloaded from Zenodo: https://zenodo.org/records/17047290.

## References

[1] NHS . Breast Screening (Mammogram) 2021 [cited 2025 Jun 24] Available from: https://www.nhs.uk/tests-and-treatments/breast-screening-mammogram/when-youll-be-invited-and-who-should-go/.

[2] Tyrer J , Duffy SW, Cuzick J. A breast cancer prediction model incorporating familial and personal risk factors. Stat Med 2004;23:1111–30. 10.1002/sim.1668. 15057881

[3] Brentnall AR , Harkness EF, Astley SM, Donnelly LS, Stavrinos P, Sampson S, . Mammographic density adds accuracy to both the Tyrer-Cuzick and Gail breast cancer risk models in a prospective UK screening cohort. Breast Cancer Res 2015;17:147. 10.1186/S13058-015-0653-5. PMC4665886 26627479

[4] National Institute for Health and Care Excellence . Familial breast cancer: classification, care and managing breast cancer and related risks in people with a family history of breast cancer. Clinical Guideline [CG164]. 2013 [cited 2022 May 31]. Available from: https://www.nice.org.uk/guidance/cg164/ifp/chapter/drug-treatment-to-reduce-the-risk-of-breast-cancer. 31940157

[5] UK National Screening Committee . Criteria for a population screening programme. GOV.UK [Internet]. 2022 [cited 2025 Jun 24]. Available from: https://www.gov.uk/government/publications/evidence-review-criteria-national-screening-programmes/criteria-for-appraising-the-viability-effectiveness-and-appropriateness-of-a-screening-programme.

[6] Wright SJ , Eden M, Ruane H, Byers H, Evans DG, Harvie M, . Estimating the cost of 3 risk prediction strategies for potential use in the United Kingdom National Breast Screening Program. MDM Policy Pract 2023;8:238146832311713. 10.1177/23814683231171363. PMC10161319 37152662

[7] Gray E , Donten A, Karssemeijer N, van Gils C, Evans DG, Astley S, . Evaluation of a stratified national breast screening program in the United Kingdom: an early model-based cost-effectiveness analysis. Value Health 2017;20:1100–9. 10.1016/j.jval.2017.04.012. 28964442

[8] French DP , McWilliams L, Payne K, Woof V, Harrison A, Jerrison M, . The feasibility of risk-stratified screening as routine practice in the NHS Breast Screening Programme: the PROCAS2 research programme including intervention development studies, large feasibility study, health economic analyses and agenda-setting meeting. Programme Grants for Applied Research. Southampton (UK): National Institute for Health and Care Research; 2025. 41337634

[9] Hill H , Kearns B, Pashayan N, Roadevin C, Sasieni P, Offman J, . The cost-effectiveness of risk-stratified breast cancer screening in the UK. Br J Cancer 2023;129:1801–9. 10.1038/s41416-023-02461-1. PMC10667489 37848734

[10] Pashayan N , Morris S, Gilbert FJ, Pharoah PDP. Cost-effectiveness and benefit-to-harm ratio of risk-stratified screening for breast cancer: a life-table model. JAMA Oncol 2018 1;4:1504–10. 10.1001/JAMAONCOL.2018.1901. PMC6230256 29978189

[11] Hill H , Roadevin C, Duffy S, Mandrik O, Brentnall A. Cost-effectiveness of AI for risk-stratified breast cancer screening. JAMA Netw Open 2024;7:e2431715. 10.1001/jamanetworkopen.2024.31715. PMC11377997 39235813

[12] Brentnall AR , Cuzick J, Buist DSM, Bowles EJA. Long-term accuracy of breast cancer risk assessment combining classic risk factors and breast density. JAMA Oncol 2018 13;4:e180174. 10.1001/jamaoncol.2018.0174. PMC6143016 29621362

[13] Husereau D , Drummond M, Augustovski F, de Bekker-Grob E, Briggs AH, Carswell C, . Consolidated Health Economic Evaluation Reporting Standards 2022 (CHEERS 2022) statement: updated reporting guidance for health economic evaluations. BMJ 2022;376:e067975. 10.1136/bmj-2021-067975. PMC8749494 35017145

[14] French DP , Astley S, Brentnall AR, Cuzick J, Dobrashian R, Duffy SW, . What are the benefits and harms of risk stratified screening as part of the NHS Breast Screening Programme? study protocol for a multi-site non-randomised comparison of BC-predict versus usual screening (NCT04359420). BMC Cancer 2020;20:570. 10.1186/S12885-020-07054-2. PMC7302349 32552763

[15] Jones K , Burns A. Unit costs of health and social care [Internet]. Canterbury (UK): PSSRU, University of Kent; 2021. 10.22024/UniKent/01.02.84818.

[16] RIVM . Breast cancer screening programme [Internet]. Utrecht (Netherlands): RIVM; 2023 [cited 2023 May 16]. Available from: https://www.rivm.nl/en/breast-cancer-screening-programme

[17] Weedon-Fekjaer H , Lindqvist BH, Vatten LJ, Aalen OO, Tretli S. Breast cancer tumor growth estimated through mammography screening data. Breast Cancer Res 2008;10:R41. 10.1186/BCR2092. PMC2481488 18466608

[18] Evans G , Stavrinos P, Dawe S, Harvie M, Wilson M, Maxwell A, . Improvement in risk prediction, early detection and prevention of breast cancer in the NHS Breast Screening Programme and Family History Clinic: a dual cohort study. 2015. 27559559

[19] Cancer Research UK . TNM staging for breast cancer [Internet]. 2026 [cited 2023 Sep 19]. Available from: https://www.cancerresearchuk.org/about-cancer/breast-cancer/stages-grades/tnm-staging

[20] Office for National Statistics . Cancer survival in England - adults diagnosed [Internet]. 2019 [cited 2022 Apr 14]. Available from: https://www.ons.gov.uk/peoplepopulationandcommunity/healthandsocialcare/conditionsanddiseases/datasets/cancersurvivalratescancersurvivalinenglandadultsdiagnosed.

[21] NHS Digital . Breast Screening Programme, England, 2023-2024. NHS England Digital [Internet]. 2025 [cited 2025 Jun 24]. Available from: https://digital.nhs.uk/data-and-information/publications/statistical/breast-screening-programme/england---2023-24/mainreport2324.

[22] Office for National Statistics . Population estimates for the UK, England and Wales, Scotland and Northern Ireland - office for national statistics [Internet]. 2019 [cited 2020 Mar 2]. Available from: https://www.ons.gov.uk/peoplepopulationandcommunity/populationandmigration/populationestimates/bulletins/annualmidyearpopulationestimates/mid2018.

[23] NHS Digital . Cancer Survival in England, cancers diagnosed 2016 to 2020, followed up to 2021 [Internet]. 2023 [cited 2025 Jun 24]. Available from: https://digital.nhs.uk/data-and-information/publications/statistical/cancer-survival-in-england/cancers-diagnosed-2016-to-2020-followed-up-to-2021.

[24] Cancer Research UK . Breast cancer incidence (invasive) statistics [Internet]. 2025 [cited 2022 Apr 14]. Available from: https://www.cancerresearchuk.org/health-professional/cancer-statistics/statistics-by-cancer-type/breast-cancer/incidence-invasive#heading-One.

[25] Kollias J , Murphy CA, Elston CW, Ellis IO, Robertson JFR, Blamey RW. The prognosis of small primary breast cancers. Eur J Cancer 1999;35:908–12. 10.1016/S0959-8049(99)00056-8. 10533470

[26] Wen J , Ye F, Li S, Huang X, Yang L, Xiao X, . The practicability of a novel prognostic index (PI) model and comparison with Nottingham prognostic index (NPI) in stage I–III breast cancer patients undergoing surgical treatment. PLoS One 2015;10:e0143537. 10.1371/JOURNAL.PONE.0143537. PMC4658156 26600129

[27] Cheng L , Al-Kaisi NK, Gordon NH, Liu AY, Gebrail F, Shenk RR. Relationship between the size and margin status of ductal carcinoma in situ of the breast and residual disease. J Natl Cancer Inst 1997;89:1356–60. 10.1093/JNCI/89.18.1356. 9308705

[28] Payne NR , Hickman SE, Black R, Priest AN, Hudson S, Gilbert FJ. Breast density effect on the sensitivity of digital screening mammography in a UK cohort. Eur Radiol 2025;35:177–87. 10.1007/s00330-024-10951-w. PMC11631811 39017933

[29] Hernandez Alava M , Pudney S, Wailoo A. Estimating EQ-5D By age and sex for the UK. Sheffield (UK): The University of Sheffield: Decision Support Unit; 2022. Available from: https://sheffield.ac.uk/nice-dsu/methods-development/estimating-eq-5d.

[30] Naik H , Howell D, Su S, Qiu X, Brown MC, Vennettilli A, . EQ-5D health utility scores: data from a comprehensive Canadian cancer centre. Patient 2017;10:105–15. 10.1007/s40271-016-0190-z. 27567613

[31] NHS England . National cost collection data publication: 2023/24. 2024 [cited 2025 Jun 24]. Available from: https://app.powerbi.com/view?r=eyJrIjoiZGQxYjNkOGUtOTIwMC00N2VjLWEyM2EtYjAzOGMwNWU5ODQ1IiwidCI6IjM3YzM1NGIyLTg1YjAtNDdmNS1iMjIyLTA3YjQ4ZDc3NGVlMyJ9.

[32] Laudicella M , Walsh B, Burns E, Smith PC. Cost of care for cancer patients in England: evidence from population-based patient-level data. Br J Cancer 2016;114:1286–92. 10.1038/BJC.2016.77. PMC4891510 27070711

[33] Office for National Statistics . Mortality rates (qx), principal projection, England and Wales - Office For National Statistics [Internet]. 2019 [cited 2020 Mar 2]. Available from: https://www.ons.gov.uk/peoplepopulationandcommunity/birthsdeathsandmarriages/lifeexpectancies/datasets/mortalityratesqxprincipalprojectionenglandandwales.

[34] Kaur MN , Yan J, Klassen AF, David JP, Pieris D, Sharma M, . A systematic literature review of health utility values. Med Decis Making 2022;42:704–19. 10.1177/0272989X211065471. PMC9189726 35042379

[35] Pourrahmat M-M , Kim A, Kansal AR, Hux M, Pushkarna D, Fazeli MS, . Health state utility values by cancer stage: a systematic literature review. Eur J Health Econ 2021;22:1275–88. 10.1007/S10198-021-01335-8. PMC8526485 34125315

[36] NHS England . 2018/19 National cost collection data publication [Internet]. 2022 Available from: https://www.england.nhs.uk/publication/2018-19-national-cost-collection-data-publication/

[37] The National Institute for Health and Care Excellence . NICE health technology evaluations: The manual. London (UK): NICE; 2022 [cited 2023 Nov 21]. Available from: https://www.nice.org.uk/process/pmg36/chapter/economic-evaluation#presentation-of-data-and-results.

[38] Evans D , McWilliams L, Astley S, Brentnall AR, Cuzick J, Dobrashian R, . Quantifying the effects of risk-stratified breast cancer screening when delivered in real time as routine practice versus usual screening: the BC-Predict non-randomised controlled study (NCT04359420). Br J Cancer 2023;128:2063–71. 10.1038/s41416-023-02250-w. PMC10066938 37005486

[39] Gilbert FJ , Payne NR, Allajbeu I, Yit L, Vinnicombe S, Lyburn I, . Comparison of supplemental breast cancer imaging techniques-interim results from the BRAID randomised controlled trial. Lancet 2025;405:1935–44. 10.1016/S0140-6736(25)00582-3. 40412427

[40] Gommers J , Hernström V, Josefsson V, Sartor H, Schmidt D, Hjelmgren A, . Interval cancer, sensitivity, and specificity comparing AI-supported mammography screening with standard double reading without AI in the MASAI study: a randomised, controlled, non-inferiority, single-blinded, population-based, screening-accuracy trial. Lancet 2026;407:505–14. 10.1016/S0140-6736(25)02464-X. 41620232

[41] Nanaa M , Manavaki R, van Nijnatten TJA, Stranz N, Carriero S, Coleman WA, . Tumour growth rate and invasive interval cancer characteristics in a UK breast cancer screening population. Eur Radiol 2025;35:4001–12. 10.1007/s00330-024-11342-x. PMC12165881 39838090

[42] MacInnes EG , Duffy SW, Simpson JA, Wallis MG, Turnbull AE, Wilkinson LS, . Radiological audit of interval breast cancers: estimation of tumour growth rates. Breast 2020;51:114–9. 10.1016/j.breast.2020.03.006. PMC7375675 32298962

[43] Carver T , Hartley S, Lee A, Cunningham AP, Archer S, Babb de Villiers C, . CanRisk tool-a web interface for the prediction of breast and ovarian cancer risk and the likelihood of carrying genetic pathogenic variants. Cancer Epidemiol Biomark Prev 2021;30:469–73. 10.1158/1055-9965.EPI-20-1319. PMC7611188 33335023

