# Supplementary material for: Implementing a Risk-Stratified National Breast Screening Programme in the United Kingdom: A Model-Based Cost-effectiveness Analysis
Source: Cancer Epidemiol Biomarkers Prev. 2026 Jul 31;35(10):1752–62. doi: 10.1158/1055-9965.EPI-26-0103 (PMC13628088; doi:10.1158/1055-9965.EPI-26-0103)
Supplement: Supplementary file 1 [file epi-26-0103_supplementary_methods_suppsm1.docx]

Supplementary Materials S1: Description of Model Stucture

*Interventions*

The exemplar stratified-NBSPs included in this analysis were informed by a programme of work looking at the feasibility of rolling out risk-stratified NBSPs into the NHS (called PROCAS-2) (1,2). The PROCAS-2 programme study sought to evaluate the feasibility of implementation of a risk-prediction strategy at a woman’s first attendance at breast cancer screening. The chosen approach, called ‘BC-PREDICT’, used an online version of the Tyrer-Cuzick risk questionnaire to capture information on women’s family history, demographic, and lifestyle risk factors(3). This information was combined with automated breast density measurements captured at the woman’s first mammography appointment using the Volpara TruScan system. Using these questionnaires and breast density measurement each women’s 10-year and lifetime breast cancer risk were estimated. The full process of identifying breast cancer risk and providing risk feedback was detailed in French et al 2020 (4).

*The stratification process*

Each individual woman in the simulation was initially assigned a Volpara breast density score, 10-year risk, and lifetime breast cancer risk based on data from a published study, BC-PREDICT where risk data were calculated for 15,613 women using updated versions of the Tyrer-Cuzick questionnaire and Volpara breast density measurement (4,5).

To account for error in the risk prediction algorithm we augment the synthetic population data with a corrected 10-year risk and corrected lifetime risk for each woman. We did this by devising a simple parametric model of the follow-up process in the Brentnall *et al.* study and using numerical optimisation to calibrate beta distributions to the expected and observed case numbers. We assigned corrected 10-year risks to the synthetic population by assuming that each individual’s corrected and uncorrected risks sit at the same quantile of their respective risk distributions; this is equivalent to assuming that for any two individuals in the population, the individual with the higher (lower) uncorrected risk always has the higher (lower) corrected risk. The resulting 10-year risk distribution should yield the same distribution of case groups by risk cohort as is observed in the 10-year follow-up data. We assigned corrected lifetime risks under the assumption that the relationship between corrected 10-year and lifetime risk is the same as that between uncorrected 10-year and lifetime risk by applying Gaussian process regression, a machine learning approach which infers functional relationships between continuous-valued data, using the R package GauPro(6).

*Cancer screening*

Based on her estimated 10-year risk, each individual woman was assigned to a pre-specified screening interval. A published study was used to define each of the different risk groups(4). In MANC-RISK-SCREEN, high-risk was defined to be a 10-year breast cancer risk of over 8% while moderate-risk was defined to fall between the values of 5% and 8%. Population-level risk was defined to be less than 5% in the Risk-1 strategy. In the Risk-3 strategy, which included less frequent screening for women at lower-risk (less than 1.5% 10-year risk), population-level risk was defined as a 10-year risk between 1.5% and 5%.

*Natural History of Cancer*

The natural history of cancer was represented using a mathematical algorithm (see the model diagrams and tumour growth model functional form choice documents in the GitHub repository) (7). A uniform random number was drawn and compared with the woman’s lifetime cancer risk to determine if she will experience a cancer in her lifetime. If cancer occurs, then the age at clinical diagnosis was drawn from an empirically-derived distribution of age at diagnosis(8). Observed distributions of age at diagnosis are by definition conditional on individuals surviving long enough to experience a diagnosis; the expected age at death due to all-cause mortality among people diagnosed with cancer is therefore higher than among the general population. To account for this, we applied a correction based on Bayes’ rule to the distribution of age at diagnosis to ensure that age at death from all-cause mortality and age at diagnosis could be drawn independently while still reflecting observed distributions (technical details are given in the documentation in the GitHub repository). A tumour growth rate was drawn and the age of tumour genesis back-calculated using the tumour-growth model (9).

At a screening event, the size of the tumour (if present) was determined using the tumour growth model. The sensitivity of screening was assumed to be conditional on the size of the tumour and the woman’s breast density. A uniform random number was drawn against this sensitivity to determine if the tumour was detected.

*Diagnosis and treatment*

When a tumour was identified by screening, or by reaching the size at which it would be clinically diagnosed, a cancer stage was assigned. MANC-RISK-SCREEN used TNM (extent of the tumour (T); extent of spread to the lymph nodes (N); presence of metastasis (M)) stages. The TNM stage was used as more data are available for this grading system rather than other systems, such as the Nottingham Prognostic Indicator (10).

*Mortality*

When a cancer was identified by screening and then diagnosed and allocated a defined stage of disease, a new age of death was drawn to reflect the impact of the stage of cancer on mortality. In MANC-RISK-SCREEN, exponential survival functions were fitted to 5-year survival for tumours of different stages (11).

Supplementary Materials S2: Model Inputs

*Cancer Incidence*

The probability that an individual would experience breast cancer in their lifetime was assumed to be equal to their predicted lifetime risk using the Tyrer-Cuzick questionnaire and Volpara automated breast density measurement. This assumption implied that the strategy to estimate each women’s risk was a perfect estimate of their risk of breast cancer. We also carried out an analysis which accounted for misclassification under the Tyrer-Cuzick questionnaire. In this case we assumed that the probability that an individual would experience breast cancer in their lifetime was equal to their corrected risk, with the expected risk acting only to influence clinical decision-making. Once it was determined whether an individual woman would experience breast cancer, an age of incidence was applied based on the age distribution of cancers provided by a study published by Cancer Research UK(8).

*Tumour growth*

The process for reflecting tumour growth assumed a continuous time model and is detailed in the documentation folder of the GitHub repository (7,12). In the absence of UK data, the chosen model was based on observations from Norwegian women, providing the closest approximation for women in the UK.

*Risk-Stratification*

Three key risk parameters were recorded for each woman: Volpara breast density; 10-year breast cancer risk as defined by the Tyrer-Cuzick questionnaire including breast density; and lifetime breast cancer risk. To allow the sharing of these data in the model files, the synthpop package was used in R to generate a synthetic dataset which maintained the structure of the underlying data (13). This process ensured that lifetime risk remained correlated to 10-year risk. In this analysis it was assumed that all women chose to have their breast cancer risk predicted and agreed to change their screening intervals. In both the base-case and misclassification analysis, it was assumed that risk-stratified screening schedules are based on expected risks rather than corrected risks; the expected risk is the one which is known to clinicians, whereas the corrected risk is not directly observable.

*Uptake of screening*

The analysis allowed for imperfect uptake (attendance) for each round of breast screening by using three parameters in MANC-RISK-SCREEN. Women were assumed to have a 62.5% chance of attending their first mammography. For subsequent screening rounds, the probability of attending was assumed to be 88.7% if the woman had attended at least one previous screening event and 20.8% if she had never attended screening. All uptake probabilities were taken from the NHS Digital annual breast cancer screening report(14).

*Sensitivity of Screening*

The sensitivity of mammography was assumed to be dependent on the density of a woman’s breasts. This assumption was made because denser breast tissue can mask tumours and therefore reduce the sensitivity of breast screening. Women were first assigned to a Volpara Breast Density group between 1 and 4 based on their breast density. To calculate the Volpara Breast Density group-specific sensitivity given the size of the tumour “the ratio of the odds of a true positive result for that Volpara Breast Density Group compared with the population average odds was combined with the odds of a true positive result given the tumour size alone” (12).

*Mammography recall rate*

Women who were assumed to attend screening and did not have a cancer present could experience a recall for further testing after receiving a false-positive result. The rate of recall was estimated using data obtained directly from the UK NBSP (14). Of the women recalled for further testing, a proportion were assumed to also have to undergo a biopsy of a suspicious lump. The probability of this biopsy occurring was included in the analysis.

*Diagnosis and treatment by cancer stage*

When a woman was assumed to have a diagnosis of a cancer this was assigned into one of three categories: invasive; non-invasive or micro-invasive; an advanced tumour according to the TNM staging system. This assignment was based on the size of the tumour at diagnosis, taken from the tumour growth model. To assign cancers of a given size to different tumour types and stages, a matrix was created using data from Kollias et al 1999, Wen et al 2015 and Cheng et al 1997 (15–17). To estimate the TNM stage of a cancer, the degree of lymph node involvement must be ascertained in addition to the cancer size. While Wen et al contains full details of the number of lymph nodes involved, Kollias et al only contains whether there was node involvement or not. As such the distribution of the number of nodes from Wen et al was applied to the data from Kollias et al to estimate the number of nodes involved for these data. When combined with data on non-invasive cancers from Cheng et al, this allows a cancer to be assigned to stage I, II, III, or non-invasive status with given probabilities based on its size. The probability of a cancer of a given size being advanced (metastatic) was taken from the NHS audit of screen-detected breast cancers (18).

Supplementary Materials S3: Full description of the approach taken to risk misclassification

The Tyrer-Cuzick model is known to give imperfect risk predictions, meaning that the incidence of cancer in specific risk cohorts is not fully consistent with the risks assigned to that cohort. A 2018 study by Brentall *et al.* followed up individuals assigned 10-year risks using the Tyrer-Cuzick questionnaire and Volpara breast density measurement after ten years and measured the number of diagnosed breast cancer cases in each risk group and found discrepancies between the expected and observed numbers of cases (19). In the updated analysis, we use the results of this study to correct for prediction error in the Tyrer-Cuzick model by calibrating parametric risk distributions to the expected and observed case numbers and then mapping between these two distributions.

The Tyrer-Cuzick model assigns each individual to one of 5 risk groups defined by bounds on that individual’s 10-year risk: <2%, 2-3%, 3-5%, 5-8%, and ≥8%. The supplementary material to Brentnall et al. 2018 provides the numbers of individuals in their study assigned to each risk group, stratified by age, along with the expected number of cases in each age-risk subgroup at time of risk stratification and the observed number of cases in each age-risk subgroup at the 10 year follow-up date. We denote the number of individuals in a given age group who are assigned to risk group $i$ by $N_{i}$ , with $N=\sum_{i} N_{i}$ giving the total number of individuals in the sample, and denote the expected and observed number of cases in risk subgroup $i$ by $E_{i}$ and $O_{i}$ respectively.

We approximate the distributions of underlying and true risks underlying this data using beta distributions with parameters chosen by simulating the process that generates $N_{i}$, $E_{i}$, and $O_{i}$. Given a beta distribution with parameters $(\alpha_{E}, \beta_{E})$, we can simulate the generation of expected case numbers as follows

1. For each individual in a population of $N$ individuals, draw a 10-year risk $\rho_{j}\sim\mathrm{Beta}(\alpha_{E}, \beta_{E})$;
2. Reorder the 10-year risks so that $\rho_{j}<\rho_{j+1}$ for $j=1,\ldots,N-1$;
3. For each individual draw a 0/1-valued cancer status $y\sim\mathrm{Bernoulli}\mathbf{(}\rho_{j})$;
4. Generate synthetic expected case numbers $\tilde{E}_{1},\ldots,\tilde{E}_{5}$ by summing the number of positive cancer statuses in each successive block of $N_{i}$ individuals, i.e.

$$\tilde{E}_{i}= \sum_{j=N_{i-1}+1}^{N_{i}} y_{j};$$

5. Carry out steps 1 to 4 1,000 times to get an estimate of the expectation of the expected number of cases in each risk group:

$$\mathbb{E[}\tilde{E}_{i}]= \frac{1}{1,000}\sum_{n=1}^{1,000} \tilde{E}_{i};$$

6. Calculate the root mean square error associated with the parameters $(\alpha_{E}, \beta_{E})$:

$$\sqrt{\sum_{i=1}^{5} {\mathbb{(E[}\tilde{E}_{i}]- E_{i})}^{2}} .$$

Using the optim function from the stats package of the R Programming Language to minimise this root mean square error as a function of $\alpha_{E}$ and $\beta_{E}$, we identify optimal underlying expected risk distribution parameters $(\hat{\alpha}_{E}, \hat{\beta}_{E})$. The same method applied to the observed case numbers gives us optimal parameters $(\hat{\alpha}_{O}, \hat{\beta}_{O})$ for the distribution of true risk.

To correct for prediction error in our synthetic population, we apply a quantile matching approach using the two fitted parameter sets, $(\hat{\alpha}_{E}, \hat{\beta}_{E})$ and $(\hat{\alpha}_{O}, \hat{\beta}_{O})$. This approach relies on the assumption that the ordering of different individual’s risks is the same under the true underlying risk distribution as under the estimated risk distribution obtained from the Tyrer-Cuzick model, so that the true risk for every individual assigned to risk group $i$ is higher than that of any individual belonging to risk group $j\leq i$ and lower than that of any individual assigned to risk group $j>i$. Given an individual with estimated lifetime risk $p_{10}$, we define the corrected lifetime risk $\hat{p}_{10}$ to be

$$\hat{p}_{10}= F_{\beta}^{-1}\left( F_{\beta}\left( p_{10} \right|\alpha_{E}, \beta_{E} \right) \left| \alpha_{O}, \beta_{O} \right) ,$$

so that an individual whose predicted risk is at the $q$^th^ percentile of the $\mathrm{Beta}\left( \alpha_{E}, \beta_{E} \right)$ distribution fit to the expected incidence data will have a corrected risk at the $q$^th^ percentile of the $\mathrm{Beta}\left( \alpha_{O}, \beta_{O} \right)$ distribution fit to the observed incidence data. This corrected risk follows the beta distribution fit to observed incidence data.

As well as 10-year risk, the Tyrer-Cuzick model estimates each individual’s lifetime risk of developing breast cancer. Because Brentnall *et al.* 2018 only includes data from a 10-year follow-up as opposed to lifetime tracking of outcomes, we can not generate a correction to lifetime risk using the same methodology as for 10-year risk. Instead, we generate a corrected lifetime risk  $\hat{p}_{L}$ given an estimated lifetime risk $p_{L}$ by assuming that the same functional relationship holds between 10-year and lifetime risk based on both estimated and corrected values, i.e. $p_{L}=f(p_{10})$ and  $\hat{p}_{L}={f(\hat{p}}_{10})$ for some function $f$. To account for noise in the relationship between risk values, we approximate this function using Gaussian process regression, a Bayesian non-parametric machine learning method which infers a probability distribution of possible functional relationships between input data and output data. Once this distribution of possible functions has been estimated, it can be used to randomly generate new datapoints. We used the R Programming Language package GauPro to infer a Gaussian process $f$ from the estimated 10-year and lifetime risks in our synthetic population. For each individual in the synthetic population, we augmented their corrected 10-year risk $\hat{p}_{10}$ with a corrected lifetime risk $\hat{p}_{L}={f(\hat{p}}_{10})$ .

Based on our 10-year and lifetime risk estimate correction methods, we generated an augmented synthetic population identical in characteristics to that used in previous iterations of the model, with two additional fields specifying a corrected 10-year risk and corrected lifetime risk. The synthetic risk data includes individuals under 50 years old and between 50-60 years old. Separate correction models were fitted for these two age groups based on the age-stratified expected and observed case numbers reported by Brentnall *et al.*, and the appropriate correction model was chosen based on each individual’s age.

Supplementary References

1.	Manchester Cancer Research Centre. Manchester Cancer Research Centre [Internet]. 2023 [cited 2023 Nov 24]. PROCAS 2. Available from: https://www.mcrc.manchester.ac.uk/impact-case-studies/procas-2/

2.	French, DP, McWilliams, L, Payne, K, Woof, V, Harrison, A, Jerrison, M, et al. The feasibility of risk-stratified screening as routine practice in the NHS Breast Screening Programme: the PROCAS2 research programme including intervention development studies, large feasibility study, health economic analyses and agenda-setting meeting. NIHR J Libr Programme Grants Appl Res. 2025.

3.	Tyrer J, Duuy SW, Cuzick J. A breast cancer prediction model incorporating familial and personal risk factors. Stat Med. 2004;23:1111–30. doi:10.1002/sim.1668

4.	French DP, Astley S, Astley S, Brentnall AR, Cuzick J, Dobrashian R, et al. What are the benefits and harms of risk stratified screening as part of the NHS breast screening Programme? Study protocol for a multi-site non-randomised comparison of BC-predict versus usual screening (NCT04359420). BMC Cancer. 2020 Jun 18;20(1):1–14. doi:10.1186/S12885-020-07054-2/TABLES/1 PubMed PMID: 32552763.

5.	Evans G, Stavrinos P, Dawe S, Harvie M, Wilson M, Maxwell A, et al. Improvement in risk prediction, early detection and prevention of breast cancer in the NHS Breast Screening Programme and Family History Clinic: a dual cohort study. 2015.

6.	Erickson C. GauPro: Gaussian Process Fitting [Internet]. 2024. Available from: https://CRAN.R-project.org/package=GauPro

7.	Anonymous until publication. MANC-RISK-SCREEN [Internet]. Available from: https://anonymous.4open.science/r/MANC-RISK-SCREEN-10EF/

8.	Cancer Research UK. Breast cancer incidence (invasive) statistics [Internet]. 2025 [cited 2022 Apr 14]. Available from: https://www.cancerresearchuk.org/health-professional/cancer-statistics/statistics-by-cancer-type/breast-cancer/incidence-invasive#heading-One

9.	Weedon-Fekjær H, Lindqvist BH, Vatten LJ, Aalen OO, Tretli S. Breast cancer tumor growth estimated through mammography screening data. Breast Cancer Res BCR. 2008 Jun 8;10(3):R41. doi:10.1186/BCR2092 PubMed PMID: 18466608.

10.	TNM staging for breast cancer [Internet]. [cited 2023 Sep 19]. Available from: https://www.cancerresearchuk.org/about-cancer/breast-cancer/stages-grades/tnm-staging

11.	Office for National Statistics. Cancer survival in England - adults diagnosed [Internet]. 2019 [cited 2022 Apr 14]. Available from: https://www.ons.gov.uk/peoplepopulationandcommunity/healthandsocialcare/conditionsanddiseases/datasets/cancersurvivalratescancersurvivalinenglandadultsdiagnosed

12.	Gray E, Donten A, Karssemeijer N, van Gils C, Evans DG, Astley S, et al. Evaluation of a Stratified National Breast Screening Program in the United Kingdom: An Early Model-Based Cost-Effectiveness Analysis. Value Health. 2017 Sep 1;20(8):1100–9. doi:10.1016/j.jval.2017.04.012 PubMed PMID: 28964442.

13.	Nowok B, Raab GM, Dibben C, Snoke J, Lissa C van. synthpop: Generating Synthetic Versions of Sensitive Microdata for Statistical Disclosure Control [Internet]. 2022 [cited 2023 Oct 6]. Available from: https://cran.r-project.org/web/packages/synthpop/index.html

14.	NHS Digital. NHS England Digital [Internet]. 2025 [cited 2025 Jun 24]. Breast Screening Programme, England, 2023-2024. Available from: https://digital.nhs.uk/data-and-information/publications/statistical/breast-screening-programme/england---2023-24/mainreport2324

15.	Kollias J, Murphy CA, Elston CW, Ellis IO, Robertson JFR, Blarney RW. The prognosis of small primary breast cancers. Eur J Cancer. 1999 Jun 1;35(6):908–12. doi:10.1016/S0959-8049(99)00056-8 PubMed PMID: 10533470.

16.	Wen J, Ye F, Li S, Huang X, Yang L, Xiao X, et al. The Practicability of a Novel Prognostic Index (PI) Model and Comparison with Nottingham Prognostic Index (NPI) in Stage I–III Breast Cancer Patients Undergoing Surgical Treatment. PLOS ONE. 2015 Nov 1;10(11):e0143537. doi:10.1371/JOURNAL.PONE.0143537 PubMed PMID: 26600129.

17.	Cheng L, Al-Kaisi NK, Gordon NH, Liu AY, Gebrail F, Shenk RR. Relationship Between the Size and Margin Status of Ductal Carcinoma In Situ of the Breast and Residual Disease. JNCI J Natl Cancer Inst. 1997 Sep 17;89(18):1356–60. doi:10.1093/JNCI/89.18.1356 PubMed PMID: 9308705.

18.	Public Health England. An audit of screen detected breast cancers for the year of screening April 2013 to March 2014. 2015.

19.	Brentnall AR, Cuzick J, Buist DSM, Bowles EJA. Long-term Accuracy of Breast Cancer Risk Assessment Combining Classic Risk Factors and Breast Density. JAMA Oncol. 2018 Sep 1;4(9):e180174. doi:10.1001/jamaoncol.2018.0174 PubMed PMID: 29621362; PubMed Central PMCID: PMC6143016.
