# Supplementary material for: Implementing a Risk-Stratified National Breast Screening Programme in the United Kingdom: A Model-Based Cost-effectiveness Analysis
Source: Cancer Epidemiol Biomarkers Prev. 2026 Jul 31;35(10):1752–62. doi: 10.1158/1055-9965.EPI-26-0103 (PMC13628088; doi:10.1158/1055-9965.EPI-26-0103)
Supplement: Supplementary file 2 [file epi-26-0103_supplementary_table_s10_suppst10.docx]

**Supplementary Table S10: Covariance matrix for survival post-BC**

| 0.08878 |  |  |
| --- | --- | --- |
| 0.01819 | 0.00373 |  |
| 0.01866 | 0.00384 | 0.00395 |
