# Supplementary material for: Implementing a Risk-Stratified National Breast Screening Programme in the United Kingdom: A Model-Based Cost-effectiveness Analysis
Source: Cancer Epidemiol Biomarkers Prev. 2026 Jul 31;35(10):1752–62. doi: 10.1158/1055-9965.EPI-26-0103 (PMC13628088; doi:10.1158/1055-9965.EPI-26-0103)
Supplement: Supplementary file 3 [file epi-26-0103_supplementary_table_s11_suppst11.docx]

**Supplementary Table S11: Covariance matrix for metastatic survival**

| 0.01157 |  |  |
| --- | --- | --- |
| 0.00884 | 0.00705 |  |
| 0.00804 | 0.00613 | 0.00560 |
