# Supplementary material for: Implementing a Risk-Stratified National Breast Screening Programme in the United Kingdom: A Model-Based Cost-effectiveness Analysis
Source: Cancer Epidemiol Biomarkers Prev. 2026 Jul 31;35(10):1752–62. doi: 10.1158/1055-9965.EPI-26-0103 (PMC13628088; doi:10.1158/1055-9965.EPI-26-0103)
Supplement: Supplementary file 5 [file epi-26-0103_supplementary_table_s13_suppst13.docx]

Supplementary Table S13: Results when using a discount rate of 1.5%

Table S13 shows the estimated costs, QALYs, and cost-effectiveness of the six strategies when the discount rate is reduced to 1.5% for both health outcomes and costs. Figure S3.1 shows the cost-effectiveness acceptability curve for this analysis.

Table S13: Costs, Outcomes, and Cost-effectiveness of Six Breast Cancer Screening Strategies
