# Supplementary material for: Implementing a Risk-Stratified National Breast Screening Programme in the United Kingdom: A Model-Based Cost-effectiveness Analysis
Source: Cancer Epidemiol Biomarkers Prev. 2026 Jul 31;35(10):1752–62. doi: 10.1158/1055-9965.EPI-26-0103 (PMC13628088; doi:10.1158/1055-9965.EPI-26-0103)
Supplement: Supplementary file 6 [file epi-26-0103_supplementary_table_s14_suppst14.docx]

Supplementary Table S14. Predicted Clinical Outcomes Under Different Screening Strategies

Table S4. provides the clinical predictions of the model under different screening strategies including the number of screen detected cancers per 1,000 women invited, number of lifetime false-positive results per 1,000 women invited, average diameter of cancers detected by screening, average life expectancy in the population and average life expectancy for women who will develop cancer.

| Strategy | Lifetime screen detected cancers per 1000 women invited to screening^1^ | Lifetime false-positive results per 1000 women invited to screening^1^ | Average tumour diameter at screen detection (mm) | Average Life expectancy for the general population^2^ | Average life expectancy for people who develop cancer^2^ |
| --- | --- | --- | --- | --- | --- |
| No Screening | 0 | 0 | N/A | 84.57 | 82.65 |
| 3 Yearly | 22.55 | 164.52 | 12.0 | 84.66 | 83.55 |
| Risk-3 | 24.53 | 171.18 | 11.8 | 84.67 | 83.66 |
| Risk-1 | 24.75 | 179.79 | 11.7 | 84.67 | 83.70 |
| 2 Yearly | 30.28 | 272.04 | 11.3 | 84.69 | 83.88 |
| Risk-2 | 33.61 | 330.08 | 10.6 | 84.70 | 84.06 |

^1^ This includes women who both attend and do not attend screening and rates may therefore differ to those observed in the screening programme

^2^ Life expectancy for women who are alive at age 50 and have not been diagnosed with a cancer prior to the start of breast screening
