# Supplementary material for: Implementing a Risk-Stratified National Breast Screening Programme in the United Kingdom: A Model-Based Cost-effectiveness Analysis
Source: Cancer Epidemiol Biomarkers Prev. 2026 Jul 31;35(10):1752–62. doi: 10.1158/1055-9965.EPI-26-0103 (PMC13628088; doi:10.1158/1055-9965.EPI-26-0103)
Supplement: Supplementary file 7 [file epi-26-0103_supplementary_table_s1_suppst1.docx]

Supplementary Table S1. -

| **Section/topic** | **Item No** | **Guidance for reporting** | **Reported in section** |
| --- | --- | --- | --- |
| **Title** | | |  |
| Title | 1 | Identify the study as an economic evaluation and specify the interventions being compared. | ___Title Page__ |
| **Abstract** | | |  |
| Abstract | 2 | Provide a structured summary that highlights context, key methods, results, and alternative analyses. | ___Page 5__ |
| **Introduction** | | |  |
| Background and objectives | 3 | Give the context for the study, the study question, and its practical relevance for decision making in policy or practice. | __Page 7__ |
| **Methods** | | |  |
| Health economic analysis plan | 4 | Indicate whether a health economic analysis plan was developed and where available. | ___N/A__ |
| Study population | 5 | Describe characteristics of the study population (such as age range, demographics, socioeconomic, or clinical characteristics). | _Page 9, 10 |
| Setting and location | 6 | Provide relevant contextual information that may influence findings. | ___Page 9__ |
| Comparators | 7 | Describe the interventions or strategies being compared and why chosen. | _Page 9, 10_ |
| Perspective | 8 | State the perspective(s) adopted by the study and why chosen. | __Page 9___ |
| Time horizon | 9 | State the time horizon for the study and why appropriate. | __Page 9___ |
| Discount rate | 10 | Report the discount rate(s) and reason chosen. | __Page 9___ |
| Selection of outcomes | 11 | Describe what outcomes were used as the measure(s) of benefit(s) and harm(s). | _Page 9, 13, __ |
| Measurement of outcomes | 12 | Describe how outcomes used to capture benefit(s) and harm(s) were measured. | __ Supplementary materials S3.3_ |
| Valuation of outcomes | 13 | Describe the population and methods used to measure and value outcomes. | _ Supplementary materials S3.3 |
| Measurement and valuation of resources and costs | 14 | Describe how costs were valued. | _Page 15, Supplementary materials S3.2_ |
| Currency, price date, and conversion | 15 | Report the dates of the estimated resource quantities and unit costs, plus the currency and year of conversion. | _Page 9, Page 15, Supplementary materials S3.2__ |
| Rationale and description of model | 16 | If modelling is used, describe in detail and why used. Report if the model is publicly available and where it can be accessed. | _Data availability statement, Supplementary materials 2, GitHub documentation “Model Summary”_ |
| Analytics and assumptions | 17 | Describe any methods for analysing or statistically transforming data, any extrapolation methods, and approaches for validating any model used. | Page 17-18__ |
| Characterizing heterogeneity | 18 | Describe any methods used for estimating how the results of the study vary for subgroups. | _N/A_ |
| Characterizing distributional effects | 19 | Describe how impacts are distributed across different individuals or adjustments made to reflect priority populations. | __N/A__ |
| Characterizing uncertainty | 20 | Describe methods to characterise any sources of uncertainty in the analysis. | __Page 17_____ |
| Approach to engagement with patients and others affected by the study | 21 | Describe any approaches to engage patients or service recipients, the general public, communities, or stakeholders (such as clinicians or payers) in the design of the study. | __Page 14, Supplementary materials 3.3__ |
| **Results** | | |  |
| Study parameters | 22 | Report all analytic inputs (such as values, ranges, references) including uncertainty or distributional assumptions. | __Table 2, 3 and 4. Supplementary materials S3.1, 3.2, 3.4, Supplementary materials 5 |
| Summary of main results | 23 | Report the mean values for the main categories of costs and outcomes of interest and summarise them in the most appropriate overall measure. | _Table 5, Table 6_ |
| Effect of uncertainty | 24 | Describe how uncertainty about analytic judgments, inputs, or projections affect findings. Report the effect of choice of discount rate and time horizon, if applicable. | _Figure 3, Supplementary materials 7.1 and 8.1 |
| Effect of engagement with patients and others affected by the study | 25 | Report on any difference patient/service recipient, general public, community, or stakeholder involvement made to the approach or findings of the study | _ Page 14, Supplementary materials 3.3_ |
| **Discussion** | | |  |
| Study findings, limitations, generalizability, and current knowledge | 26 | Report key findings, limitations, ethical or equity considerations not captured, and how these could affect patients, policy, or practice. | _Page 24__ |
| **Other relevant information** | | | |
| Source of funding | 27 | Describe how the study was funded and any role of the funder in the identification, design, conduct, and reporting of the analysis | __Funding declaration__ |
| Conflicts of interest | 28 | Report authors conflicts of interest according to journal or International Committee of Medical Journal Editors requirements. | _Author conflict of interest statement__ |
