# Supplementary material for: Implementing a Risk-Stratified National Breast Screening Programme in the United Kingdom: A Model-Based Cost-effectiveness Analysis
Source: Cancer Epidemiol Biomarkers Prev. 2026 Jul 31;35(10):1752–62. doi: 10.1158/1055-9965.EPI-26-0103 (PMC13628088; doi:10.1158/1055-9965.EPI-26-0103)
Supplement: Supplementary file 8 [file epi-26-0103_supplementary_table_s2_suppst2.docx]

Supplementary Table S2: Clinical parameters in deterministic model

| **Parameter** | **Value** | **Updated or added since Gray model?** | **Sources and Assumptions** |  |  |  |
| --- | --- | --- | --- | --- | --- | --- |
| Probability of attending a screening appointment | 62.5% for the first screen  88.7% if one previous screen attended  20.8% if first screen not attended | Yes | NHS Digital 2025 (1) |  |  |  |
| Uptake for prediction of risk of breast cancer | 100% in base case | Yes | Expert opinion |  |  |  |
| Uptake for receipt of risk information | 100% in base case | Yes | Expert opinion |  |  |  |
| Proportion of women who change screening intervals based on risk information | 100% in base case | Yes | Expert opinion |  |  |  |
| Age of death due to all-cause mortality | ~weibull(9.38,89.35) | Yes | Office for National Statistics (2)  Using the probability of dying at a given age, a small Markov model was created with a starting age of 50. This Markov model contained the states alive and dead and recorded the number of individuals expected to die at each age. From this, a simulated data set was created containing the age of death for each individual in the cohort. Using the fitdistrplus package in R, a Weibull distribution was fit to this data. |  |  |  |
| Survival for stage 1 breast cancer | exp(-5.462) | Yes | NHS Digital 2023 (3)  Exponential survival curve fitted for median survival for women with stage 1 cancer at 5 years post-diagnosis. A previous version of this model had used Nottingham Prognostic Indicator grade rather than stage but found an exponential curve to be the best fit of multiple models selected. As such the same model was applied to data related to cancer stage |  |  |  |
| Survival for stage 2 breast cancer | exp(-4.023) | Yes | NHS Digital 2023 (3)  Exponential survival curve fitted for median survival for women with stage 2 cancer at 5 years post-diagnosis. A previous version of this model had used Nottingham Prognostic Indicator grade rather than stage but found an exponential curve to be the best fit of multiple models selected. As such the same model was applied to data related to cancer stage |  |  |  |
| Survival for stage 3 breast cancer | exp(-2.465) | Yes | NHS Digital 2023 (3)  Exponential survival curve fitted for median survival for women with stage 3 cancer at 5 years post-diagnosis. A previous version of this model had used Nottingham Prognostic Indicator grade rather than stage but found an exponential curve to be the best fit of multiple models selected. As such the same model was applied to data related to cancer stage |  |  |  |
| Survival for stage 4 breast cancer for women aged up to 54 | exp(-1.787) | Yes | Office for National Statistics 2019 (4)  Exponential survival curve fitted for median survival for women aged under 54 with stage 4 cancer at 5 years post-diagnosis. A previous version of this model had used Nottingham Prognostic Indicator grade rather than stage but found an exponential curve to be the best fit of multiple models selected. As such the same model was applied to data related to cancer stage  Note: newer data does not break down metastatic survival by age so figures from 2019 continue to be used |  |  |  |
| Survival for stage 4 breast cancer for women aged 55 to 74 | exp(-1.388) | Yes | Office for National Statistics 2019 (4)  Exponential survival curve fitted for median survival for women aged 55 to 74 with stage 4 cancer at 5 years post-diagnosis. As this age group covered two age bands in the underlying data, a weighted average of the median 5-year survival was calculated. A previous version of this model had used Nottingham Prognostic Indicator grade rather than stage but found an exponential curve to be the best fit of multiple models selected. As such the same model was applied to data related to cancer stage  Note: newer data does not break down metastatic survival by age so figures from 2019 continue to be used |  |  |  |
| Survival for stage 4 breast cancer for women aged over 75 | exp(-1.011) | Yes | Office for National Statistics 2019 (4)  Exponential survival curve fitted for median survival for women aged over 75 with stage 4 cancer at 5 years post-diagnosis. A previous version of this model had used Nottingham Prognostic Indicator grade rather than stage but found an exponential curve to be the best fit of multiple models selected. As such the same model was applied to data related to cancer stage  Note: newer data does not break down metastatic survival by age so figures from 2019 continue to be used |  |  |  |
| Breast cancer incidence by age | See Supplementary Materials 2 | Yes | Cancer Research UK 2025 (5) |  |  |  |
| 10 year risk of breast cancer estimated using the Tyrer-Cuzick version 8 and Volpara breast density group | See Supplementary Materials 2 | Yes | Observations of breast density (using Volpara TruDensity), 10 year breast cancer risk (using Tyrer-Cuzick version 8 and Volpara TruDensity), and lifetime risk (using Tyrer-Cuzick version 8 and Volpara TruDensity) for 15,613 women were shared securely by the BC-PREDICT research team. In order to allow this input data to be shared outside the research team, a synthetic data set was created using the synthpop package in R. This approach resulted in a data set with 15,613 synthetically generated observations of breast density, 10 year risk, and lifetime risk. The approach also preserves the structure of the underlying data including correlations. |  |  |  |
| Lifetime risk of breast cancer estimated using the Tyrer-Cuzick version 8 and Volpara breast density group | See Supplementary Materials 2 | Yes | Observations of breast density (using Volpara TruDensity), 10 year breast cancer risk (using Tyrer-Cuzick version 8 and Volpara TruDensity), and lifetime risk (using Tyrer-Cuzick version 8 and Volpara TruDensity) for 15,613 women were shared securely by the BC-PREDICT research team. In order to allow this input data to be shared outside the research team, a synthetic data set was created using the synthpop package in R. This approach resulted in a data set with 15,613 synthetically generated observations of breast density, 10 year risk, and lifetime risk. The approach also preserves the structure of the underlying data including correlations. |  |  |  |
| Volpara breast density estimate | See Supplementary Materials 2 | Yes | Observations of breast density (using Volpara TruDensity), 10 year breast cancer risk (using Tyrer-Cuzick version 8 and Volpara TruDensity), and lifetime risk (using Tyrer-Cuzick version 8 and Volpara TruDensity) for 15,613 women were shared securely by the BC-PREDICT research team. In order to allow this input data to be shared outside the research team, a synthetic data set was created using the synthpop package in R. This approach resulted in a data set with 15,613 synthetically generated observations of breast density, 10 year risk, and lifetime risk. The approach also preserves the structure of the underlying data including correlations. |  |  |  |
| Probability that a cancer is metastatic given the age of the woman | 25=4.62%  35=8.67%  45=10.98%  55=12.71%  65=14.25%  75=15.98%  85=17.30% | No | Age nearest to woman’s current age at diagnosis of cancer used |  |  |  |
| Probability of cancer being diagnosed as stage I, II, or III given size of tumour | See Supplementary Materials 2 | Yes | Kollias et al. (1998), Wen et al. (2015), Cheng et al. (1997) (6–8)  The supplementary data provided in Wen et al. provided observations of cancer size, number of lymph node involvements, and other variables for 1,661 women. Given that none of the patients had metastatic cancer, it was possible to determine the stage of cancer for each woman using the size of the tumour and number of lymph nodes involved. This data was used to determine the probability that cancers of different sizes would be of different stages. However, the data set contained few observation for women with very small tumours. As such, data from Kollias et al. was combined with that included in Wen et al. to provide a better representation of the distribution of stages in very small cancers. As Kollias et al. only included whether lymph nodes were involved or not and did not state the number of nodes, it was assumed that half of patients with node involvement had one node and half had more than one node. The distributions of stages across cancer size was compared between the studies and aside from the smallest cancers the studies showed close agreement.  The likelihood a cancer of a given size was a DCIS was calculated using the distribution of DCIS tumour sizes from Cheng et al (1997) and the proportion of cancers that were DCIS (21.1%) from NHS England screening statistics. The stage_by_size_mat was re-calculated such that a cancer of a given size had a probability of being of stage I-III or a DCIS. |  |  |  |
| Mammographic sensitivity by Volpara Density Group (VDG) | VDG1=75.0%  VDG2=73.5%%  VDG3=59.8%  VDG4=51.3%  Average=66.6% | Yes | Payne et al. (2025) (9) |  |  |  |
| Recall (false-positive) rate for screening | 3.1% | Yes | NHS Digital 2025 (1) |  |  |  |
| Biopsy rate for recalls | 50.6% | Yes | NHS Digital 2025 (1) |  |  |  |
| Growth Model Parameters |  |  |  |  |  |  |
| Mean tumour doublings at clinical detection | 6.5 | No | Weedon-Fekjær et al. (2008) (12) |  |  |  |
| Standard deviation of tumour doublings at clinical detection | 0.535 | No | Weedon-Fekjær et al. (2008) (12) |  |  |  |
| Log normal mean of tumour growth rate | | | | 1.07 | No | Weedon-Fekjær et al. (2008) (12) |
| Log normal standard deviation of tumour growth rate | 1.31 | No | Weedon-Fekjær et al. (2008) (12) |  |  |  |
| Maximum tumour size (mm) | 128 | No | Weedon-Fekjær et al. (2008) (12) |  |  |  |
| Starting tumour size (mm) | 0.25 | No | Weedon-Fekjær et al. (2008) (12) |  |  |  |
