# Supplementary material for: Implementing a Risk-Stratified National Breast Screening Programme in the United Kingdom: A Model-Based Cost-effectiveness Analysis
Source: Cancer Epidemiol Biomarkers Prev. 2026 Jul 31;35(10):1752–62. doi: 10.1158/1055-9965.EPI-26-0103 (PMC13628088; doi:10.1158/1055-9965.EPI-26-0103)
Supplement: Supplementary file 9 [file epi-26-0103_supplementary_table_s3_suppst3.docx]

Supplementary Table S3: Costs used in deterministic model

| **Parameter** | **Value** | **Updated or added since Gray model?** | **Sources and Assumptions** |
| --- | --- | --- | --- |
| Cost of risk stratification | £7.40 | Yes | Wright et al (2023) (13)  Costs in underlying microcosting updated to current values |
| Cost of mammography screen | £39.93 | Yes | Pragmatic microcosting  See model parameter update document |
| Cost of follow up | £130.97 | Yes | Inflated from value in Gray et al. (2017) (11) |
| Cost of biopsy | £558 | Yes | NHS England (2024) (14) |
| Cost of ultrasound screening | £102 | Yes | NHS England (2024) (14) |
| Cost of MRI screening | £162 | Yes | NHS England (2024) (14) |
| Cost of treating breast cancer by stage, age, and time lived with cancer | See Supplementary Materials 2 | Yes | Laudicella et al. (2015) (15)  Laudicella provide estimates of the costs of treating breast cancer in the UK from the two years preceding to eight years following diagnosis. Costs are broken down by stage of cancer (early versus late) and age (under or over 65). These costs were included as an input table in the model. They were then inflated to 2024 levels. An exponential regression was then fit to the data to predict the total cost of treating an individual’s cancer given the length of time they had the cancer, their age of diagnosis, and the stage of their cancer. If a patient was alive for over 8 years following diagnosis the cost of treating their cancer was assumed to be £0 in any subsequent years. |
| Cost of treating a ductal carcinoma in-situ | £12,346.83 | Yes | Inflated from value in Gray et al. (2017) (11) |
