# Supplementary material for: Implementing a Risk-Stratified National Breast Screening Programme in the United Kingdom: A Model-Based Cost-effectiveness Analysis
Source: Cancer Epidemiol Biomarkers Prev. 2026 Jul 31;35(10):1752–62. doi: 10.1158/1055-9965.EPI-26-0103 (PMC13628088; doi:10.1158/1055-9965.EPI-26-0103)
Supplement: Supplementary file 10 [file epi-26-0103_supplementary_table_s4_suppst4.docx]

Supplementary Table S4: Utility values used in the deterministic model

| **Parameter** | **Value** | **Updated or added since Gray model?** | **Sources and Assumptions** |
| --- | --- | --- | --- |
| Age-related utility values for healthy individuals | See Supplementary Materials 2 | Yes | Hernandez Alava et al (2022) (16) |
| Utility early breast cancer in the first year | 0.82 | Yes | Naik et al. (2017) (17)  Potential utility values were identified from two systematic reviews of utility values in breast cancer (18,19). From the studies included in these reviews, three studies which estimated utility values covering local and metastatic cancer, conducted in high income countries, and using either EQ-5D and relevant value sets or the time-trade off approach were identified. A focus group was then held with three women who had recovered from breast cancer to decide which of the three sets of utility values they felt best represented the experience of women in the UK.  The values in the chosen study did not change between the first and subsequent years and it was for this reason that the women in the focus group chose the set. However, the ability to choose different values for subsequent years was retained in the model in case future research suggests this is useful for inclusion. |
| Utility early breast cancer in subsequent years | 0.82 | Yes | Naik et al. (2017) (17)  Potential utility values were identified from two systematic reviews of utility values in breast cancer (18,19). From the studies included in these reviews, three studies which estimated utility values covering local and metastatic cancer, conducted in high income countries, and using either EQ-5D and relevant value sets or the time-trade off approach were identified. A focus group was then held with three women who had recovered from breast cancer to decide which of the three sets of utility values they felt best represented the experience of women in the UK.  The values in the chosen study did not change between the first and subsequent years and it was for this reason that the women in the focus group chose the set. However, the ability to choose different values for subsequent years was retained in the model in case future research suggests this is useful for inclusion. |
| Advanced breast cancer in the first year | 0.75 |  | Naik et al. (2017) (17)  Potential utility values were identified from two systematic reviews of utility values in breast cancer (18,19). From the studies included in these reviews, three studies which estimated utility values covering local and metastatic cancer, conducted in high income countries, and using either EQ-5D and relevant value sets or the time-trade off approach were identified. A focus group was then held with three women who had recovered from breast cancer to decide which of the three sets of utility values they felt best represented the experience of women in the UK.  The values in the chosen study did not change between the first and subsequent years and it was for this reason that the women in the focus group chose the set. However, the ability to choose different values for subsequent years was retained in the model in case future research suggests this is useful for inclusion. |
| Advanced breast cancer in subsequent years | 0.75 | Yes | Naik et al. (2017) (17)  Potential utility values were identified from two systematic reviews of utility values in breast cancer (18,19). From the studies included in these reviews, three studies which estimated utility values covering local and metastatic cancer, conducted in high income countries, and using either EQ-5D and relevant value sets or the time-trade off approach were identified. A focus group was then held with three women who had recovered from breast cancer to decide which of the three sets of utility values they felt best represented the experience of women in the UK.  The values in the chosen study did not change between the first and subsequent years and it was for this reason that the women in the focus group chose the set. However, the ability to choose different values for subsequent years was retained in the model in case future research suggests this is useful for inclusion. |
