# Supplementary material for: Implementing a Risk-Stratified National Breast Screening Programme in the United Kingdom: A Model-Based Cost-effectiveness Analysis
Source: Cancer Epidemiol Biomarkers Prev. 2026 Jul 31;35(10):1752–62. doi: 10.1158/1055-9965.EPI-26-0103 (PMC13628088; doi:10.1158/1055-9965.EPI-26-0103)
Supplement: Supplementary file 11 [file epi-26-0103_supplementary_table_s9_suppst9.docx]

Supplementary Table 9: Distributions used in the probablistic analysis

Supplementary Table 9: Input parameters with sampling distributions and hyperparameters used in PSA

| **Parameter** | **Distribution** | **Hyperparameter 1** | **Hyperparameter 2** | **Updated or added since Gray model?** |
| --- | --- | --- | --- | --- |
| **Mammographic Sensitvity** | | **Mean** | **Standard deviation** |  |
| Beta 1 | Normal | 1.47 | 0.1 | No |
| Beta 2 | Normal | 6.51 | 0.5 | No |
| **VDG modifiers** | | $\alpha$ | $\beta$ |  |
| VDG 1 | Beta | 96 | 16 | Yes |
| VDG 2 | Beta | 298 | 86 | Yes |
| VDG 3 | Beta | 212 | 93 | Yes |
| VDG 4 | Beta | 61 | 39 | Yes |
| **Growth rate distribution** | | **Mean** | **Standard deviation** |  |
| $\alpha_{1}$ | Normal | 1.07 | 0.09 | No |
| $\alpha_{2}$ | Normal | 1.31 | 0.11 | No |
|  |  |  |  |  |
| **Survival post-BC** | | **Mean** | **Correlated draws** |  |
| $\gamma$ stage I | Multivariate normal (MVN) | -5.46 | See Table 5 | Yes |
| $\gamma$ stage II | MVN | -3.82 | See Table 5 | Yes |
| $\gamma$ stage III | MVN | -2.72 | See Table 5 | Yes |
| **Survival metastatic BC** |  | **Mean** | **Correlated draws** |  |
| $\gamma$ stage IV, age <55 | MVN | -1.79 | See Table 6 | Yes |
| $\gamma$ stage IV, age 55-74 | MVN | -1.39 | See Table 6 | Yes |
| $\gamma$ stage IV, age >74 | MVN | -1.01 | See Table 6 | Yes |
| **Utility weights** | | | |  |
| Early | 1-exp(MVN) | -1.71 | See Table 7 | Yes |
| Advanced cancer | 1-exp(MVN) | -1.39 | See Table 7 | Yes |
| **Costs** |  | **Mean** | **Standard Deviation** |  |
| Risk stratification | Log normal | 2.13 | 0.06 | Yes |
| Cost multiplier (independently drawn and applied for each other cost item) | Normal | 0 | 0.102 | Yes |
